# Breast cancer risk factors in relation to molecular subtypes in breast cancer patients from Kenya

**DOI:** 10.1186/s13058-021-01446-3

**Published:** 2021-06-26

**Authors:** Shahin Sayed, Shaoqi Fan, Zahir Moloo, Ronald Wasike, Peter Bird, Mansoor Saleh, Asim Jamal Shaikh, Jonine D. Figueroa, Richard Naidoo, Francis W. Makokha, Kevin Gardner, Raymond Oigara, Faith Wambui Njoroge, Pumza Magangane, Miriam Mutebi, Rajendra Chauhan, Sitna Mwanzi, Dhirendra Govender, Xiaohong R. Yang

**Affiliations:** 1 Department of Pathology, Aga Khan University, Nairobi, Kenya; 2 University of Cape Town, Cape Town, South Africa; 3 National Cancer Institute of the National Institutes of Health (NCI/NIH) Bethesda, Maryland Rockville, USA; 4 AIC Kijabe Hospital, Kijabe, Kenya; 5 The University of Edinburgh, Edinburgh, UK; 6 Mount Kenya University, Thika, Kenya; 7 Columbia University Medical Centre, New York, USA; 8 St. Mary’s Mission Hospital, Nairobi, Kenya; 9 Kisii University, Kisii, Kenya; 10 Nyeri Provincial General Hospital, Nyeri, Kenya; 11 University of the Witwatersrand, Johannesburg, South Africa; 12 PathCare, Cape Town, South Africa

**Keywords:** Breast cancer, Molecular subtypes, Risk factors, Kenya, Sub-Saharan Africa

## Abstract

**Background:**

Few studies have investigated risk factor heterogeneity by molecular subtypes in indigenous African populations where prevalence of traditional breast cancer (BC) risk factors, genetic background, and environmental exposures show marked differences compared to European ancestry populations.

**Methods:**

We conducted a case-only analysis of 838 pathologically confirmed BC cases recruited from 5 groups of public, faith-based, and private institutions across Kenya between March 2012 to May 2015. Centralized pathology review and immunohistochemistry (IHC) for key markers (ER, PR, HER2, EGFR, CK5-6, and Ki67) was performed to define subtypes. Risk factor data was collected at time of diagnosis through a questionnaire. Multivariable polytomous logistic regression models were used to determine associations between BC risk factors and tumor molecular subtypes, adjusted for clinical characteristics and risk factors.

**Results:**

The median age at menarche and first pregnancy were 14 and 21 years, median number of children was 3, and breastfeeding duration was 62 months per child. Distribution of molecular subtypes for luminal A, luminal B, HER2-enriched, and triple negative (TN) breast cancers was 34.8%, 35.8%, 10.7%, and 18.6%, respectively. After adjusting for covariates, compared to patients with ER-positive tumors, ER-negative patients were more likely to have higher parity (OR = 2.03, 95% CI = (1.11, 3.72), *p* = 0.021, comparing ≥ 5 to ≤ 2 children). Compared to patients with luminal A tumors, luminal B patients were more likely to have lower parity (OR = 0.45, 95% CI = 0.23, 0.87, p = 0.018, comparing ≥ 5 to ≤ 2 children); HER2-enriched patients were less likely to be obese (OR = 0.36, 95% CI = 0.16, 0.81, *p* = 0.013) or older age at menopause (OR = 0.38, 95% CI = 0.15, 0.997, *p* = 0.049). Body mass index (BMI), either overall or by menopausal status, did not vary significantly by ER status. Overall, cumulative or average breastfeeding duration did not vary significantly across subtypes.

**Conclusions:**

In Kenya, we found associations between parity-related risk factors and ER status consistent with observations in European ancestry populations, but differing associations with BMI and breastfeeding. Inclusion of diverse populations in cancer etiology studies is needed to develop population and subtype-specific risk prediction/prevention strategies.

**Supplementary Information:**

The online version contains supplementary material available at 10.1186/s13058-021-01446-3.

## Background

Women in Africa have lower incidence rates of breast cancer (BC) than women in developed countries (age-standardized rates (ASR) per 100,000 of 36 vs. 74), but higher mortality rates (ASR of 17 vs. 15) [1]. Furthermore, there is variation in the relative survival (RS) from BC by stage and country-level human development index (HDI) in sub-Saharan Africa (SSA) with the 5-year RS after breast cancer diagnosis in Mauritius at 83.2% and the lowest in Uganda at 12.1%, while it ranges between 40.1 and 64% in Kenya as per data abstracted from the Eldoret and Nairobi Cancer Registries, respectively [2]. Furthermore, survival differences in SSA remain for any given breast cancer stage with the lowest 3-year breast cancer-specific survival observed in Nigeria at 38% compared with 68% in Black women from Namibia, thus underlying as yet unexplained risks with survival [3]. In Kenya, country figures indicate that BC is the most frequently diagnosed cancer among women, representing 20.8% of all cancer cases, and the second most common cause from cancer mortality [4].

Although advanced stage at presentation, lack of awareness about BC and limited access to available screening and treatment options [5] are contributing factors to disparate mortality rates, whether incidence for more aggressive breast cancers are higher in African women remains controversial. Women of African descent present with BCs a decade earlier than their Caucasian counterparts [6, 3], and despite correcting for risk factor distribution, their tumors still tend to be estrogen receptor (ER) negative [7], suggesting the interplay of other biologic and genetic differences that remain largely unexplored.

Breast cancer can be divided into several molecular subtypes based on gene expression profiling analysis, which are subsequently corroborated by a panel of immunohistochemical (IHC) markers including ER, progesterone receptor (PR), human epidermal growth receptor factor 2 (HER2), proliferation marker Ki-67, cytokeratin (CK) 5/6, and epidermal growth factor receptor (EGFR). Epidemiologic studies have demonstrated that BC risk associated with established risk factors, including genetic and environment/lifestyle factors, differ for different breast cancer subtypes [8], which highlights the importance of developing subtype-specific risk prediction and prevention strategies [9]. Overwhelmingly, these breast cancer prediction models have been derived from European ancestry women and some studies have noted poor performance in African women [10]. This is likely explained by the differential associations of risk factors such as parity and obesity for ER-positive and ER-negative cancers and higher frequencies of ER-negative cancers among African women. In addition, the prevalence of breast cancer risk factors, including genetic background and environmental exposures, show marked differences between indigenous African and European and even African American women. Notably, women in African countries are more likely to have high exposures to infectious agents (malaria and other parasites), and a low prevalence of traditional BC risk factors (including low or late parity, lack of breastfeeding, obesity, and exogenous hormone use), which may contribute to differences in the risk of different BC subtypes. Furthermore, there are great variations in genetic structure and exposures as well as breast cancer subtype distributions across different African populations [11, 7, 12]. Therefore, studies in diverse indigenous African populations will allow for a broader capture of associations between risk factors and tumor subtypes, particularly for exposures and subtypes that are in general very rare but are prevalent in African populations. Findings from these studies will improve our understanding of risk factor heterogeneity and our ability to develop risk prediction models that are better tailored for specific African populations.

Here, in this study, using carefully annotated risk factor and pathology data collected from 838 BC patients enrolled from multiple hospitals across Kenya, we aimed to evaluate distributions of established BC risk factors across BC subtypes.

## Methods

### Study population and risk factor data

The study has been previously described but in brief, 838 pathologically confirmed BC cases were collected across Kenya between March 2012 and May 2015 [13]. There were 15 hospital/health facilities which we grouped into 5 network/regional facilities: Aga Khan University (AKU) hospitals (including AKU hospitals at Kisumu, Mombasa, and Nairobi), AIC Kijabe Hospital, Nyeri Provincial General Hospital (PGH), St Mary’s Mission Hospital (Nairobi), and others (Supplementary Table 1). The grouping was based on whether public, faith-based or private institutions. Institutional ethics approval was obtained. Socio-demographic, clinical, reproductive, and known breast cancer risk factor data were collected using a standardized questionnaire.

### Pathology, immunohistochemical data, and molecular subtypes

Pathologic characteristics including histologic grade, histologic tumor type, tumor size, lymph node stage, lymphovascular invasion, and ER/PR/HER2 status were extracted from the clinical database. Central pathology review and IHC for ER/PR/HER2 of all breast carcinoma tissue were done at AKU Hospital, Nairobi, and interpreted by SS and ZM. AKU Pathology department is a College of American Pathologists accredited laboratory and as such enrolls in proficiency testing schemes for breast biomarkers. Additional slides were cut at 5 μm and subjected to IHC stains for EGFR, CK5/6, and Ki67 (Dako Monoclonal mouse anti-human antibodies were used; wild type EGFR polyclonal antibody in a dilution of 1:200, CK5/6 clone D5/16 B4 ready to use, Ki-67 Clone MIB-1, ready to use) according to the manufacturer specifications as previously described [13], with appropriate control tissues included, and stained on the DAKO Autostainer link instrument.

ER and PR tumor expression were considered positive by IHC with ≥ 1% nuclear staining. HER2 expression was determined by IHC and fluorescence in situ hybridization (FISH), the latter in case of an equivocal HER2 IHC result. An IHC score of 3+ or a FISH-positive test result was defined as HER2-positive [14]. Ki-67 was considered high if 20% or more of the cells showed nuclear staining based on St Gallen recommendation [15].

We used Ki-67 status (low/high) to discriminate luminal A and B and used tumor grade as a surrogate for patients with missing Ki-67 [16]. For EGFR and CK5/6, a result was considered positive for any amount of cytoplasmic or membranous staining in any percentage of tumor cells as per the recommendations from the British Columbia study for defining the Basal subtype of breast cancer [17].

**Fig. 1:**
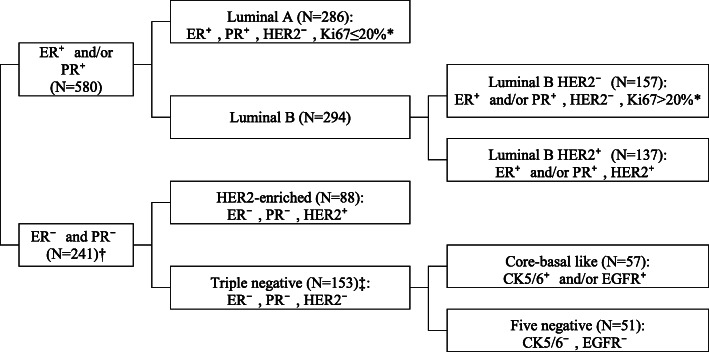
Breast tumor subtype definition in Kenyan breast cancer patients (N=838). *Tumor grade was used to determine tumor subtypes in the absence of ki67: if tumor grade is low or intermediate, define tumor subtype as “Luminal A”; if tumor grade is high, define tumor subtype as “Luminal B HER2^-^”. †Seventeen cases are not included due to their missing HER2 status. ‡Forty-five cases are not included due to their missing CK5/6 and EGFR status. CK5/6, cytokeratin 5/6; EGFR, epidermal growth factor receptor; ER, estrogen receptor; HER2, human epidermal growth factor receptor-2; PR, progesterone receptor

Molecular subtypes were defined based on previous clinically validated guidelines [18] (Fig. 1): luminal A: ER+ and/or PR+, HER2−, and low Ki-67/histologic grade (I or II); luminal B-HER2+: ER+ and/or PR+, and HER2+; luminal B-high proliferative: ER+ or PR+, HER2−, and high Ki-67/histologic grade (III); HER2-enriched: ER−, PR−, and HER2+; and triple-negative (TN): ER−, PR−, HER2 (Fig. 1). Due to the small sample size, in primary subtype analysis, we grouped the two luminal B subtypes into a single subtype for risk factor associations. For patients with EGFR and CK5/6 data available, we further stratified TN patients into core-basal like (CK5/6+ and/or EGFR+) and five negative (CK5/6− and EGFR−).

### Statistical analysis

Distributions of breast cancer risk factors, including sociodemographic, reproductive, and tumor pathologic characteristics in the overall study population and by hospital groups, were assessed using the chi-squared test or Fisher’s exact test. Multivariable polytomous logistic regression models were used to determine associations between BC risk factors and tumor molecular subtypes (ER status or luminal A-like as the reference).

All regression models were fully adjusted for the same covariates (except for where noted): age at diagnosis, BMI, age at menarche, age at first pregnancy, number of children, averaged breastfeeding duration, age at menopause, family history of breast cancer in 1st degree female relatives, highest education level, and occupation. A two-tailed P value less than 0.05 was considered statistically significant. All analyses were performed with SAS v9.4 statistical software (SAS Institute Inc.).

## Results

### Descriptive analysis of sociodemographic and reproductive characteristics

There were 838 invasive breast cancer cases with complete data on ER and PR status after exclusion of DCIS cases (n=21) and cases without any data for tumor subtype (n=8). Fifty-four percent of patients were diagnosed under 50 years of age, 69% had BMI ≥ 25 kg/m^2^ at diagnosis and 61% lived in rural areas. Our study population was also characteristic for late age at menarche (≥ 13 years, 92%), young age at first pregnancy (< 25 years, 70%), having 3 or more children (68%), high prevalence in breastfeeding (95%), and long breastfeeding duration (≥ 1 year per child, 80%) (Table 1).

**Table 1:** Distributions of breast cancer risk factors in Kenyan breast cancer patients, overall and by hospitals (*N*=838)

			Hospitals										
	Overall (***n***=838)		AKU (***n***=350, 42%)		Kijabe (***n***=105, 13%)		Nyeri (***n***=110, 13%)		St Mary’s (***n***=122, 15%)		Others (***n***=151, 18%)		
	N	%	N	%	N	%	N	%	N	%	N	%	P*
**Demographic**													
**Age at diagnosis/year**													
20–29	32	3.8	17	4.9	2	1.9	2	1.9	5	4.1	6	4.0	0.11
30–39	177	21.2	64	18.3	23	21.9	22	20.4	34	28.1	34	22.5	
40–49	242	29.0	100	28.7	37	35.2	34	31.5	35	28.9	36	23.8	
50–59	211	25.3	105	30.1	26	24.8	23	21.3	19	15.7	38	25.2	
≥ 60	172	20.6	63	18.1	17	16.2	27	25.0	28	23.1	37	24.5	
Mean (SD)	49.2 (12.8)	49.0 (11.2)	48.4 (11.3)	50.5 (13.5)	48.7 (15.1)	49.7 (12.9)							
Median (IQR)	48 (39, 57)	49 (40, 56)	47 (41, 57)	49 (41, 60)	45 (37, 58)	49 (39, 59)							
Missing	4		1		0		2		1		0		
**BMI/ kg/m**^**2**^													
Normal (< 25.0)	210	31.3	67	21.3	35	40.2	25	39.7	36	40.4	47	40.2	**< 0.0001**
Overweight (25.0–29.9)	264	39.3	126	40.0	33	37.9	28	44.4	37	41.6	40	34.2	
Obese (≥ 30.0)	197	29.4	122	38.7	19	21.8	10	15.9	16	18.0	30	25.6	
Missing	167		35		18		47		33		34		
**Family history of breast cancer in first-degree female relatives**													
No	773	92.2	314	89.7	100	95.2	99	90.0	119	97.5	141	93.4	**0.036**
Yes	65	7.8	36	10.3	5	4.8	11	10.0	3	2.5	10	6.6	
**Occupation**													
Farmer	253	30.3	47	13.4	43	41.0	82	74.5	39	32.5	42	27.8	**< 0.0001**
Employed worker	195	23.3	138	39.4	16	15.2	5	4.5	10	8.3	26	17.2	
Trader	155	18.5	66	18.9	24	22.9	2	1.8	35	29.2	28	18.5	
Housewife	156	18.7	68	19.4	19	18.1	8	7.3	27	22.5	34	22.5	
Casual worker	32	3.8	11	3.1	3	2.9	4	3.6	8	6.7	6	4.0	
Other	45	5.4	20	5.7	0	0.0	9	8.2	1	0.8	15	9.9	
Missing	2		0		0		0		2		0		
**Highest education level**													
None	257	30.7	54	15.4	41	39.0	44	40.0	57	46.7	61	40.4	**< 0.0001**
Primary	163	19.5	40	11.4	22	21.0	29	26.4	36	29.5	36	23.8	
Secondary	209	24.9	99	28.3	24	22.9	31	28.2	21	17.2	34	22.5	
Tertiary	209	24.9	157	44.9	18	17.1	6	5.5	8	6.6	20	13.2	
**Place of residence**													
Rural	511	61.0	173	49.4	79	75.2	99	90.0	65	53.3	95	62.9	**< 0.0001**
Urban	327	39.0	177	50.6	26	24.8	11	10.0	57	46.7	56	37.1	
**Exposure to smoking†**													
Never exposed	477	56.9	248	70.9	63	60.0	39	35.5	63	51.6	64	42.4	**< 0.0001**
Exposed	361	43.1	102	29.1	42	40.0	71	64.5	59	48.4	87	57.6	
**Alcohol use**													
No	760	90.7	304	86.9	98	93.3	109	99.1	115	94.3	134	88.7	**0.0009**
Yes	78	9.3	46	13.1	7	6.7	1	0.9	7	5.7	17	11.3	
**Ethnicity**													
Bantu	656	78.3	266	76.0	92	87.6	109	99.1	106	86.9	83	55.0	**< 0.0001**
Nilote	141	16.8	65	18.6	5	4.8	0	0.0	10	8.2	61	40.4	
Cushite/Mixed	41	4.9	19	5.4	8	7.6	1	0.9	6	4.9	7	4.6	
**Reproductive**													
**Age at menarche/year**													
9–12	68	8.5	30	8.6	10	11.1	7	6.9	7	5.8	14	10.0	0.40
13–14	340	42.4	150	42.9	31	34.4	39	38.2	61	50.8	59	42.1	
15–20	394	49.1	170	48.6	49	54.4	56	54.9	52	43.3	67	47.9	
Missing	36		0		15		8		2		11		
**Age at first pregnancy/year**													
Nulliparous‡	37	4.5	23	6.6	4	3.9	2	1.9	2	1.8	6	4.0	**< 0.0001**
< 20	216	26.3	62	17.7	29	28.4	34	31.8	38	34.2	53	35.3	
20–24	361	44.0	142	40.6	41	40.2	54	50.5	52	46.8	72	48.0	
25–29	150	18.3	90	25.7	21	20.6	14	13.1	14	12.6	11	7.3	
≥ 30	56	6.8	33	9.4	7	6.9	3	2.8	5	4.5	8	5.3	
Missing	18		0		3		3		11		1		
**Number of children**													
Nulliparous‡	44	5.3	26	7.4	6	5.7	2	1.8	2	1.6	8	5.3	**0.0001**
1 or 2	226	27.0	110	31.4	25	23.8	25	22.7	34	27.9	32	21.2	
3 or 4	307	36.6	135	38.6	43	41.0	47	42.7	34	27.9	48	31.8	
≥ 5	261	31.2	79	22.6	31	29.5	36	32.7	52	42.6	63	41.7	
**Cumulative breastfeeding duration/month**													
Nulliparous‡	37	4.6	23	6.6	4	4.0	2	1.9	2	1.7	6	4.2	**0.013**^**a**^
Never breastfed	7	0.9	3	0.9	2	2.0	0	0.0	0	0.0	2	1.4	
Q1: 1−< 39	191	23.5	82	23.7	27	27.0	23	21.3	32	27.8	27	18.9	
Q2: 39–< 62	192	23.6	100	28.9	28	28.0	24	22.2	16	13.9	24	16.8	
Q3: 62–< 96	182	22.4	80	23.1	17	17.0	28	25.9	24	20.9	33	23.1	
Q4: ≥ 96	203	25.0	58	16.8	22	22.0	31	28.7	41	35.7	51	35.7	
Missing	26		4		5		2		7		8		
**Averaged breastfeeding duration per child/month**													
Nulliparous‡	37	4.6	23	6.6	4	4.0	2	1.9	2	1.7	6	4.2	**0.0036**^**a**^
Never breastfed	7	0.9	3	0.9	2	2.0	0	0.0	0	0.0	2	1.4	
< 12	120	14.8	53	15.3	23	23.0	8	7.4	18	15.7	18	12.6	
12–23	407	50.1	153	44.2	46	46.0	71	65.7	64	55.7	73	51.0	
≥ 24	241	29.7	114	32.9	25	25.0	27	25.0	31	27.0	44	30.8	
Missing	26		4		5		2		7		8		
**Number of children and cumulative breastfeeding duration**													
Nulliparous or ≤3 children and < 62 months	354	43.60	179	51.7	44	44.0	43	39.8	44	38.3	44	30.8	**< 0.0001**
≤ 3 children and ≥ 62 months	79	9.73	43	12.4	5	5.0	10	9.3	8	7.0	13	9.1	
≥ 4 children and < 62 months	73	8.99	29	8.4	17	17.0	6	5.6	6	5.2	15	10.5	
≥ 4 children and ≥ 62 months	306	37.69	95	27.5	34	34.0	49	45.4	57	49.6	71	49.7	
Missing	26		4		5		2		7		8		
**Age at first pregnancy and number of children**													
Nulliparous	44	5.36	26	7.4	6	5.8	2	1.9	2	1.8	8	5.3	**< 0.0001**
Age 25+ years, 1–3 births	154	18.76	98	28.0	16	15.5	13	12.1	13	11.7	14	9.3	
Age < 25 years, 1–3 births	244	29.72	101	28.9	27	26.2	38	35.5	37	33.3	41	27.3	
Age 25+ years, 4+ births	49	5.97	23	6.6	12	11.7	4	3.7	6	5.4	4	2.7	
Age < 25 years, 4+ births	330	40.20	102	29.1	42	40.8	50	46.7	53	47.7	83	55.3	
Missing	17		0		2		3		11		1		
**Menopausal status**													
Premenopausal	438	52.4	183	52.3	64	61.0	50	45.5	67	55.4	74	49.3	0.18
Postmenopausal	398	47.6	167	47.7	41	39.0	60	54.5	54	44.6	76	50.7	
Missing	2		0		0		0		1		1		
**Age at menopause/year**^**b**^													
< 50	191	57.4	91	55.8	18	62.1	28	59.6	20	71.4	34	51.5	0.45^b^
≥ 50	142	42.6	72	44.2	11	37.9	19	40.4	8	28.6	32	48.5	
Missing	65		4		12		13		26		10		
**Cumulative hormonal contraception exposure/month**													
< 48	216	45.9	88	46.3	25	44.6	26	40.0	27	34.6	50	61.0	**0.014**
48	255	54.1	102	53.7	31	55.4	39	60.0	51	65.4	32	39.0	
Missing	367		160		49		45		44		69		

* *P* values were computed from chi-square tests except where noted. *P* values less than 0.05 are shown in bold font. ^a^ Nulliparous women and parous women who never breastfed were grouped together in chi-square test. ^b^Chi-square test was performed restricted to postmenopausal women. † Only 3.58% (*n*=30) of study participants reported ever having smoked or used smokeless tobacco. Exposure to smoking is summarized here as exposed/never exposed, where exposed is defined as personal use of tobacco as well as exposure to smoke at the workplace or home during child or adulthood. ‡ Nulliparous cases were women who reported never pregnant, never given birth, and had no children (*N*=37, 4.4%). AKU, Aga Khan University; BMI, body mass index; IQR, interquartile range; Q, quartile; SD, standard deviation.

Compared to patients admitted to the other 4 hospital groups, AKU patients were more likely to be overweight or obese (79%), have tertiary education level (45%), start the first pregnancy ≥ 25 years (35%), have < 3 children (39%), and have shorter breastfeeding duration per child, which is as expected given that AKU is a private health facility, and compared to the others, patients are generally from a higher socioeconomic status.

### Distributions of tumor subtypes and pathologic characteristics in the overall study population and by hospitals

The distribution of tumor subtypes defined by IHC markers is presented in Fig. 1 and Table 2. Overall, 69.5%, 59.4 %, and 27.4% of patients were ER+, PR+, and HER2+, respectively. After classifying BC into molecular subtypes, 34.8%, 35.8%, 10.7%, and 18.6% of patients had luminal A, luminal B, HER2-enriched, and TN breast cancers, respectively. More than 90% of patients had tumors larger than 2 cm (2–< 5 cm, 53.5%; ≥ 5 cm, 38.9%) and had intermediate-to-high tumor grade (intermediate, 45.9%; high, 49.1%). Sixty-one percent of tumors showed lymphovascular invasion. Nearly half of patients received definitive surgery, either lumpectomy or mastectomy, among which 91% had stage II or higher disease and for those cases with lymph node metastases, 39.5% were positive for extra-nodal extension. AKU patients were more likely to have small (≤ 2 cm) and early-stage tumors (*P* < 0.01). Patients admitted to Kijabe and Nyeri hospitals had higher proportions of tumors with lymphovascular invasion: 71.4% and 69.1%, respectively. There was no statistical difference in distributions of patient molecular subtypes (defined by ER, PR, and HER2) across hospitals (*P* = 0.08).

**Table 2:** Distributions of tumor characteristics in Kenyan breast cancer patients, overall and by hospitals (*N*=838)

Tumor characteristic	Overall (***n***=838)		Hospitals										
			AKU (***n***=350, 42%)		Kijabe (***n***=105, 13%)		Nyeri (***n***=110, 13%)		St Mary’s (***n***=122, 15%)		Others (***n***=151, 18%)		
	N	***%***	N	%	N	%	N	%	N	%	N	%	P*
***Tumor subtypes***													
**ER status**													
Negative	256	30.6	101	28.9	25	23.8	45	40.9	32	26.2	53	35.1	**0.029**
Positive	582	69.5	249	71.1	80	76.2	65	59.1	90	73.8	98	64.9	
**PR status**													
Negative	340	40.6	138	39.4	36	34.3	50	45.5	48	39.3	68	45.0	0.36
Positive	498	59.4	212	60.6	69	65.7	60	54.5	74	60.7	83	55.0	
**HER2 status**													
Negative	596	72.6	246	71.3	75	72.1	81	76.4	91	74.6	103	71.5	0.84
Positive	225	27.4	99	28.7	29	27.9	25	23.6	31	25.4	41	28.5	
Missing	17		5		1		4		0		7		
**Tumor molecular subtype**													
Luminal A^**a**^	286	34.8	121	35.1	46	44.2	37	34.9	40	32.8	42	29.2	0.080
Luminal B	294	35.8	127	36.8	33	31.7	26	24.5	52	42.6	56	38.9	
HER2-enriched	88	10.7	37	10.7	11	10.6	14	13.2	12	9.8	14	9.7	
Triple negative	153	18.6	60	17.4	14	13.5	29	27.4	18	14.8	32	22.2	
Missing	17		5		1		4		0		7		
Luminal A^**a**^	286	36.9	121	36.7	46	48.9	37	37.4	40	33.9	42	31.1	0.094
Luminal B - HER2^**-**^	157	20.2	65	19.7	15	16.0	15	15.2	33	28.0	29	21.5	
Luminal B - HER2^+^	137	17.7	62	18.8	18	19.1	11	11.1	19	16.1	27	20.0	
HER2-enriched	88	11.3	37	11.2	11	11.7	14	14.1	12	10.2	14	10.4	
Core-basal like	57	7.3	24	7.3	2	2.1	13	13.1	7	5.9	11	8.1	
Five negative	51	6.6	20	6.4	2	2.1	9	9.1	7	5.9	12	8.9	
Missing	62^b^		20		11		11		4		16		
***Tumor pathology***													
**Surgery**													
Core biopsy only	435	51.9	157	44.9	26	24.8	46	41.8	73	59.8	133	88.1	**< 0.0001**
Lumpectomy or mastectomy	403	48.1	193	55.1	79	75.2	64	58.2	49	40.2	18	11.9	
**Tumor size (cm)**													
< 2	41	7.6	29	13.1	8	8.1	2	2.3	1	1.9	1	1.3	**< 0.0001**
2–< 5	287	53.5	134	60.4	53	53.5	39	44.8	26	50.0	35	45.5	
≥ 5	209	38.9	59	26.6	38	38.4	46	52.9	25	48.1	41	53.3	
Missing^c^	301		128		6		23		70		74		
**Tumor overall grade**													
Grade 1 (low)	35	5.0	20	7.3	4	4.4	6	5.7	2	1.7	3	2.8	0.17
Grade 2 (intermediate)	319	45.9	130	47.3	34	37.8	44	41.5	59	50.9	52	48.2	
Grade 3 (high)	341	49.1	125	45.5	52	57.8	56	52.8	55	47.4	53	49.1	
Missing/not applicable	143		75		15		4		6		43		
**Lymphovascular invasion**													
No	326	38.9	139	39.7	30	28.6	34	30.9	47	38.5	76	50.3	**0.0029**
Yes	512	61.1	211	60.3	75	71.4	76	69.1	75	61.5	75	49.7	
Among cases with lumpectomy or mastectomy (*n*=403):													
**Tumor stage**													
Stage 0, i	30	8.2	21	12.1	2	2.6	6	10.3	1	2.4	0	0.0	**0.0040**
Stage ii	154	42.3	85	49.1	30	39.5	20	34.5	12	29.3	7	43.8	
Stage iii, iv	180	49.5	67	38.7	44	57.9	32	55.2	28	68.3	9	56.3	
Missing	39		20		3		6		8		2		
**Lymph nodes with metastasis**													
No	162	40.2	89	46.1	23	29.1	22	34.4	20	40.8	8	44.4	0.96
Yes	241	59.8	104	53.9	56	70.9	42	65.6	29	59.2	10	55.6	
**Extranodal extension**													
No	244	60.6	132	68.4	39	49.4	35	54.7	26	53.1	12	66.7	**0.022**
Yes	159	39.5	61	31.6	40	50.6	29	45.3	23	46.9	6	33.3	

* *P* values were computed from chi-square test except where noted. P values less than 0.05 are shown in bold font. ^a^ Seventy-four cases, who had missing data for both ki67 and tumor grade, were grouped into the subcategory “Luminal A” in tumor molecular subtype. ^b^Sixty-two cases whose tumor molecular subtype cannot be determined: 17 cases are due to their missing HER2 status; the other 45 cases are due to their missing CK5/6 and EGFR status. ^c^ Ninety-eight percent of missingness are from cases with core biopsy only. AKU, Aga Khan University; CK5/6, cytokeratin 5/6; EGFR, epidermal growth factor receptor; ER, estrogen receptor; HER2, human epidermal growth factor receptor-2; PR, progesterone receptor

### Associations between breast cancer risk factors and tumor subtypes ER, PR, and HER2

Results of adjusted associations between risk factors and ER status are shown in

**Table 3.** Associations between breast cancer risk factors and ER status in Kenyan breast cancer patients (*N*=838)

	ER^**+**^ ***N***=582		ER^**-**^ ***N***=256		ER^**-**^ vs. ER+	
	N	%	N	%	OR (95% CI)†	***P†***
**Age at diagnosis/year**						
20–39	155	26.8	54	21.1	1.00 (Ref)	
40–49	166	28.7	76	29.7	1.38 (0.82, 2.32)	0.23
50–59	138	23.9	73	28.5	1.20 (0.51, 2.83)	0.68
≥ 60	119	20.6	53	20.7	0.84 (0.30, 2.35)	0.74
Trend‡					0.995 (0.72, 1.39)	0.98
**BMI/ kg/m**^**2**^						
Normal (< 25.0)	136	29.8	74	34.6	1.00 (Ref)	
Overweight (25.0–29.9)	185	40.5	79	36.9	0.77 (0.49, 1.21)	0.26
Obese (≥ 30.0)	136	29.8	61	28.5	0.84 (0.51, 1.38)	0.49
Trend‡					0.92 (0.71, 1.18)	0.49
**Premenopausal: BMI**^**a**^						
Normal (< 25.0)	89	34.6	41	39.8	1.00 (Ref)	
Overweight (25.0–29.9)	102	39.7	30	29.1	0.58 (0.31, 1.10)	0.09
Obese (≥ 30.0)	66	25.7	32	31.1	0.91 (0.46, 1.77)	0.77
Trend‡					0.93 (0.66, 1.31)	0.69
**Postmenopausal: BMI**^**a**^						
Normal (< 25.0)	47	23.6	33	29.7	1.00 (Ref)	
Overweight (25.0–29.9)	83	41.7	49	44.1	1.07 (0.55, 2.08)	0.85
Obese (≥ 30.0)	69	34.7	29	26.1	0.77 (0.37, 1.61)	0.49
Trend‡					0.87 (0.61, 1.26)	0.47
**Age at menarche/year**						
≤ 13 (9–13)	141	25.4	57	23.2	1.00 (Ref)	
14	139	25.0	71	28.9	1.57 (0.94, 2.61)	0.083
≥ 15 (15–20)	276	49.6	118	48.0	1.29 (0.81, 2.04)	0.28
Trend‡					1.10 (0.88, 1.37)	0.41
**Age at first pregnancy/year**						
< 20	130	22.9	86	34.1	1.00 (Ref)	
20–24	253	44.5	108	42.9	0.71 (0.44, 1.15)	0.16
25–29	115	20.2	35	13.9	0.58 (0.30, 1.11)	0.10
Nulliparous^b^ or age ≥30	70	12.3	23	9.1	1.14 (0.48, 2.70)	0.76
Trend‡					0.92 (0.71, 1.20)	0.54
**Parity**						
Nulliparous^b^	37	6.4	7	2.7	0.36 (0.10, 1.32)	0.12
Parous	545	93.6	249	97.3	1.00 (Ref)	
**Number of children**						
1–2	171	31.4	55	22.1	1.00 (Ref)	
3–4	217	39.8	90	36.1	1.43 (0.86, 2.36)	0.16
≥ 5	157	28.8	104	41.8	**2.03 (1.11, 3.72)**	**0.021**
Trend‡					**1.43 (1.05, 1.93)**	**0.021**
**Cumulative breastfeeding duration/month**^**c**^						
Q1: 1–< 39	152	28.8	39	16.2	1.00 (Ref)	
Q2: 39–< 62	127	24.1	65	27.0	**2.38 (1.33, 4.24)**	**0.0033**
Q3: 62–< 96	124	23.5	58	24.1	1.44 (0.74, 2.80)	0.28
Q4: ≥ 96	124	23.5	79	32.8	1.58 (0.76, 3.30)	0.22
Trend‡					1.10 (0.87, 1.39)	0.43
**Mean breastfeeding duration per child/month**						
< 12	84	15.9	36	14.9	1.00 (Ref)	
12–23	277	52.6	130	53.9	1.10 (0.62, 1.94)	0.74
≥ 24	166	31.5	75	31.1	1.25 (0.68, 2.30)	0.48
Trend‡					1.12 (0.83, 1.51)	0.45
**Age at first pregnancy and number of children**						
Age 25+ years, 1–3 births	117	22.0	37	15.0	1.00 (Ref)	
Age < 25 years, 1–3 births	173	32.6	71	28.9	1.20 (0.68, 2.12)	0.53
Age 25+ years, 4+ births	34	6.4	15	6.1	1.47 (0.60, 3.61)	0.40
Age < 25 years, 4+ births	207	39.0	123	50.0	1.69 (0.93, 3.05)	0.085
Trend‡					1.19 (0.99, 1.43)	0.063
**Number of children and cumulative breastfeeding duration**						
Nulliparous or ≤ 3 children and < 62 months	265	47.0	89	35.9	1.00 (Ref)	
≤ 3 children and ≥ 62 months	56	9.9	23	9.3	1.09 (0.56, 2.09)	0.81
≥ 4 children and < 62 months	51	9.0	22	8.9	1.52 (0.74, 3.09)	0.25
≥ 4 children and ≥ 62 months	192	34.0	114	46.0	1.44 (0.89, 2.34)	0.14
Trend‡					1.14 (0.97, 1.33)	0.12
**Menopausal status**^**a**^						
Premenopausal	317	54.6	121	47.5	1.00 (Ref)	
Postmenopausal	264	45.4	134	52.5	1.44 (0.74, 2.81)	0.28
**Age at menopause/year**^**d**^						
< 50	117	21.8	74	31.5	1.00 (Ref)	
> 50	102	19.0	40	17.0	0.73 (0.40, 1.36)	0.32

† Point estimates and 95% confidence intervals were from multivariable models, adjusting for the same series of covariates (except where noticed): age at diagnosis, BMI, age at menarche, age at first pregnancy, number of children, mean breastfeeding duration per child, age at menopause, family history of breast cancer in first-degree female relative, occupation, education level, and location of facility. Estimates of numbers of children, cumulative and averaged breastfeeding duration, and combined age at first pregnancy and number of children were computed among parous women. ‡ Results were from the trend analysis using the categorical risk factor as a trend. ^a^Multivariable modeling analysis without adjusting for age at menopause. ^b^Women who reported never pregnant, never gave birth, and had no child were grouped as “Nulliparous” in modeling analyses. ^c^ Multivariable modeling analysis without adjusting for mean breastfeeding duration per child. ^d^ Multivariable Modeling analysis was restricted to postmenopausal women. BMI, body mass index; CI, confidence interval; ER, estrogen receptor; OR, odds ratio; Q, quartile

Table 3. Compared to ER-positive patients, ER-negative patients were more likely to have higher parity (OR = 2.03, 95% CI = 1.11, 3.72, P_trend_ = 0.021, comparing ≥ 5 to ≤ 2 children). ER-negative patients were also more likely to have longer cumulative breastfeeding duration (OR = 2.38, 95% CI = 1.33, 4.24; comparing ≥ 62 to < 39 months); however, these positive associations became insignificant after adjusting for a number of children. In fact, analyzing parity and breastfeeding variables together showed that the association was driven by parity (Table 3). In addition, the average duration of breastfeeding per child did not vary significantly by ER. Overall, we observed similar associations for PR to those for ER (Supplementary Table 2). BMI, either overall or by menopausal status, did not significantly vary by ER or PR status. When stratified by HER2 status, we found that, compared to HER2-negative patients, HER2-positive patients were less likely to be obese (OR = 0.58, 95% CI = 0.34, 0.97, P_trend_ = 0.038), especially among postmenopausal women (OR = 0.26, 95% CI = 0.10, 0.62, P_trend_ = 0.0026) (Supplementary Table 2). Similar results were observed when we restricted to early-stage patients (OR = 0.76, 95% CI = 0.59, 0.98, P_trend_ = 0.038) suggesting that the association was unlikely to be due to the reverse causation.

**Fig. 2:**
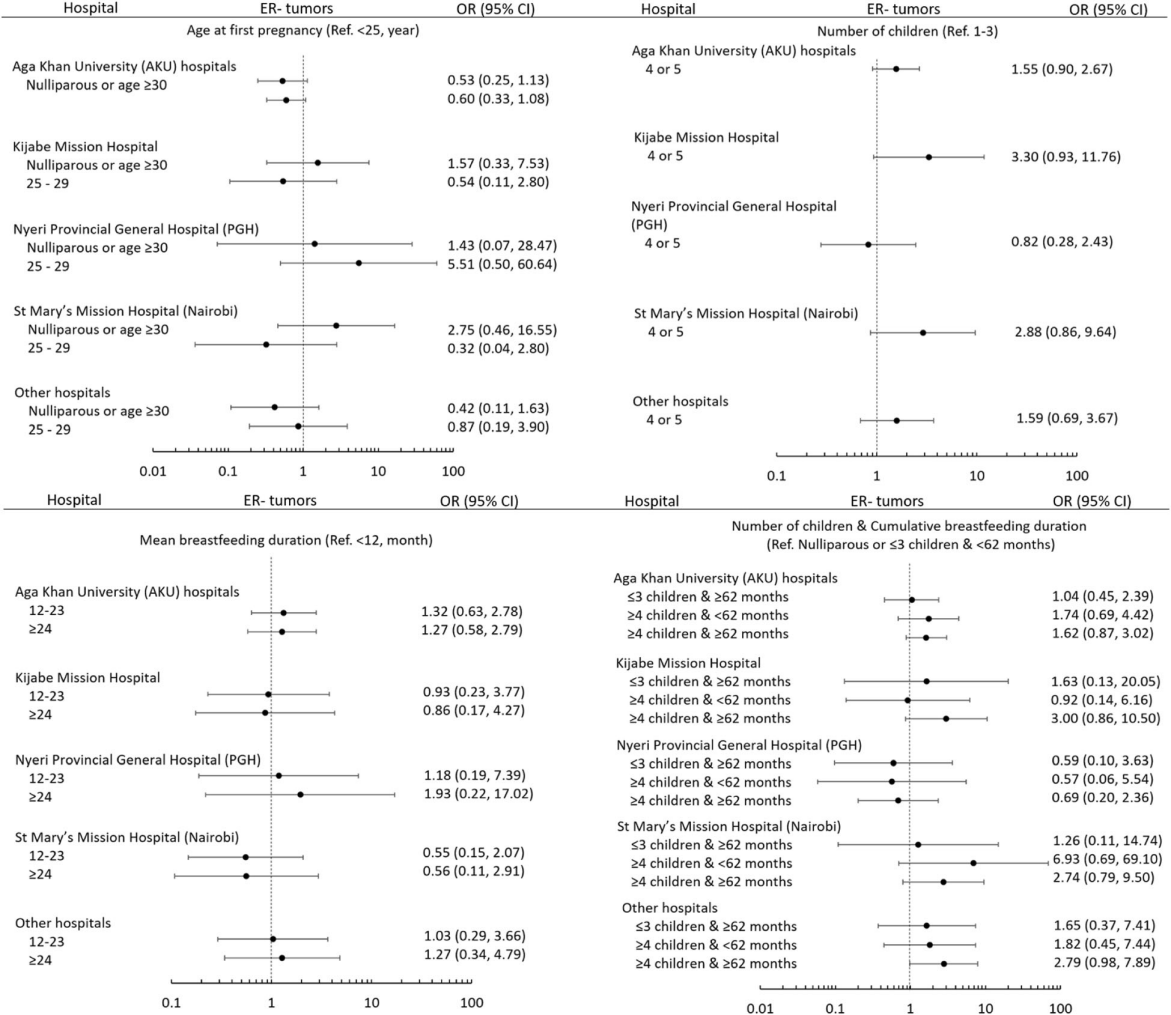
Associations between key breast cancer risk factors and ER status by hospitals. Odds ratios (OR) and 95% confidence interval (CI) were calculated from multivariable logistic regression models with ER status as the outcome variable (ER+ as reference) adjusting for categorized age at diagnosis and BMI

Given that several risk factors and clinical variables varied by hospital groups (Tables 1 and 2), we next tested whether the observed associations varied among patients admitted to different hospital groups. In this analysis, we selected five key risk factors (i.e., BMI, age at first pregnancy, number of children, and mean breastfeeding duration per child, combined number of children and cumulative breastfeeding duration) and stratified their associations with ER or HER2 (for BMI) status by five hospital groups (Fig. 2 and Supplementary Figure 1; Supplementary Table 3 and 4). With the exception of Nyeri, the associations with ER were fairly consistent across other hospitals for age at first birth, parity, and breastfeeding (Fig. 2). In contrast, the association between BMI and HER2 appeared to be driven by AKU patients (Supplementary Figure 1), among whom obesity was significantly more prevalent than patients in other hospitals; however, this pattern was also observed among patients at Kijabe Hospital.

We further evaluated the associations between the risk factors and ER in younger (< 50 years) and older (≥ 50 years) women separately. In general, the associations with most risk factors were similar in younger and older women, except that we observed an association between older age at menarche and ER-negative patients in older (OR = 2.25, 95% CI = 1.04, 4.84, P = 0.038, comparing ≥ 15 to ≤ 13 years) but not in younger women (OR = 0.98, 95% CI = 0.52, 1.87, P = 0.96, comparing ≥ 15 to ≤ 13 years) (Supplementary Table 5).

### Associations between breast cancer risk factors and molecular subtypes

Table 4 shows that the associations between BC risk factors and molecular subtypes defined by joint receptor status. Compared to luminal A patients, luminal B patients (combining luminal B-HER2+ and luminal B-high proliferative) were more likely to have lower parity (patients with 3 or 4 children, OR = 0.47, 95% CI = 0.28, 0.79, *p* = 0.005; with 5 or more children, OR = 0.45, 95% CI = 0.23, 0.87, p = 0.018, comparing to patients with 1 or 2 children). HER2-enriched patients were less likely to be obese (OR = 0.36, 95% CI = 0.16, 0.81, *p* = 0.013, comparing ≥ 30 to < 25 kg/m^2^) or to have older age at menopause (OR = 0.38, 95% CI = 0.15, 0.997, *p* = 0.049, comparing ≥ 50 to < 50 years). The HER2-BMI association appeared to be stronger among postmenopausal women (OR = 0.24, 95% CI = 0.07, 0.081, *p* = 0.022) than among premenopausal women. Overall, cumulative or average breastfeeding duration did not vary significantly across subtypes. When looking at a number of children and breastfeeding or age at first birth jointly, it appears that luminal B patients with four or more children seemed to have shorter cumulative breastfeeding duration and later age at birth compared with luminal A patients (Table 4). Further stratifying luminal B and TN subtypes did not reveal additional associations (Supplementary Table 6).

**Table 4:** Associations between breast cancer risk factors and tumor molecular subtypes in Kenyan breast cancer patients (*N*=821*)

							Tumor subtypes							
	Luminal A ***n***=286		Luminal B ***n***=294		Luminal B vs. Luminal A		HER2-enriched ***n***=88		HER2-enriched vs. Luminal A		Triple negative ***n***=153		Triple negative vs. Luminal A	
	N	%	N	%	OR (95% CI)†	***P†***	N	%	OR (95% CI)†	***P†***	N	%	OR (95% CI)†	***P†***
**Age at diagnosis/year**														
< 50	142	50.2	180	61.4	1.00 (Ref)		44	50.0	1.00 (Ref)		77	50.3	1.00 (Ref)	
≥ 50	141	49.8	113	38.6	0.87 (0.36, 2.06)	0.74	44	50.0	1.84 (0.57, 5.92)	0.30	76	49.7	0.52 (0.20, 1.40)	0.20
**BMI/ kg/m**^**2**^														
Normal (< 25.0)	60	27.3	71	30.5	1.00 (Ref)		29	41.4	1.00 (Ref)		43	32.6	1.00 (Ref)	
Overweight (25.0–29.9)	84	38.2	99	42.5	1.09 (0.66, 1.82)	0.73	25	35.7	0.55 (0.28, 1.11)	0.10	49	37.1	0.91 (0.49, 1.68)	0.75
Obese (≥ 30.0)	76	34.5	63	27.0	0.76 (0.43, 1.33)	0.33	16	22.9	**0.36 (0.16, 0.81)**	**0.013**	40	30.3	0.89 (0.46, 1.72)	0.73
Trend‡					0.87 (0.66, 1.15)	0.32			**0.59 (0.39, 0.88)**	**0.011**			0.94 (0.68, 1.32)	0.73
**Premenopausal: BMI**^**a**^														
Normal (< 25.0)	36	32.4	51	34.7	1.00 (Ref)		16	45.7	1.00 (Ref)		23	38.3	1.00 (Ref)	
Overweight (25.0–29.9)	43	38.7	59	40.1	0.98 (0.51, 1.88)	0.95	11	31.4	0.52 (0.19, 1.40)	0.20	16	26.7	0.49 (0.20, 1.21)	0.12
Obese (≥ 30.0)	32	28.8	37	25.2	0.78 (0.37, 1.61)	0.49	8	22.9	0.44 (0.14, 1.35)	0.15	21	35.0	0.84 (0.34, 2.07)	0.71
Trend‡					0.89 (0.62, 1.27)	0.52			0.65 (0.37, 1.1)	0.13			0.91 (0.57, 1.45)	0.69
**Postmenopausal: BMI**^**a**^														
Normal (< 25.0)	24	22.0	20	23.5	1.00 (Ref)		13	37.1	1.00 (Ref)		20	27.8	1.00 (Ref)	
Overweight (25.0–29.9)	41	37.6	40	47.1	1.35 (0.57, 3.20)	0.50	14	40.0	0.48 (0.17, 1.40)	0.18	33	45.8	1.70 (0.68, 4.26)	0.25
Obese (≥ 30.0)	44	40.4	25	29.4	1.10 (0.43, 2.82)	0.84	8	22.9	**0.24 (0.07, 0.81)**	**0.022**	19	26.4	1.28 (0.48, 3.46)	0.62
Trend‡					1.02 (0.64, 1.64)	0.92			**0.47 (0.26, 0.88)**	**0.018**			1.10 (0.68, 1.78)	0.71
**Age at menarche/year**														
≤ 13 (9–13)	69	25.8	71	24.8	1.00 (Ref)		23	26.4	1.00 (Ref)		33	22.9	1.00 (Ref)	
14	68	25.4	69	24.1	1.28 (0.73, 2.25)	0.39	26	29.9	1.65 (0.73, 3.74)	0.23	37	25.7	1.43 (0.73, 2.81)	0.30
≥ 15 (15–20)	131	48.9	146	51.1	1.29 (0.79, 2.13)	0.31	38	43.7	1.32 (0.64, 2.73)	0.46	74	51.4	1.43 (0.78, 2.60)	0.24
Trend‡					1.13 (0.88, 1.45)	0.34			1.12 (0.78, 1.59)	0.54			1.18 (0.88, 1.58)	0.28
**Age at first pregnancy/year**														
< 20	72	26.2	58	19.9	1.00 (Ref)		31	35.6	1.00 (Ref)		51	34.0	1.00 (Ref)	
20–24	121	44.0	133	45.5	1.51 (0.84, 2.70)	0.17	30	34.5	0.64 (0.29, 1.37)	0.25	69	46.0	0.81 (0.43, 1.51)	0.51
25–29	51	18.5	62	21.2	1.76 (0.85, 3.65)	0.13	16	18.4	0.92 (0.34, 2.51)	0.87	19	12.7	0.67 (0.28, 1.60)	0.37
Nulliparous^b^ or ≥ 30	31	11.3	39	13.4	1.06 (0.40, 2.82)	0.90	10	11.5	1.18 (0.31, 4.53)	0.81	11	7.3	0.66 (0.20, 2.18)	0.50
Trend‡					1.09 (0.82, 1.45)	0.54			1.02 (0.67, 1.56)	0.93			0.84 (0.59, 1.20)	0.33
**Parity**														
Nulliparous^b^	14	4.9	20	6.8	1.09 (0.30, 3.88)	0.90	4	4.5	0.56 (0.07, 4.77)	0.60	3	2.0	0.67 (0.11, 4.12)	0.66
Parous	272	95.1	274	93.2	1.00 (Ref)		84	95.5	1.00 (Ref)		150	98.0	1.00 (Ref)	
**Number of children**														
1–2	73	26.8	101	36.9	1.00 (Ref)		19	22.6	1.00 (Ref)		31	20.7	1.00 (Ref)	
3–4	113	41.5	101	36.9	**0.47 (0.28, 0.79)**	**0.005**	26	31.0	0.85 (0.37, 1.98)	0.71	59	39.3	1.16 (0.59, 2.29)	0.66
≥ 5	86	31.6	72	26.3	**0.45 (0.23, 0.87)**	**0.018**	39	46.4	1.71 (0.65, 4.53)	0.28	60	40.0	1.27 (0.56, 2.89)	0.56
Trend‡					**0.65 (0.47, 0.90)**	**0.0099**			1.34 (0.82, 2.20)	0.24			1.11 (0.75, 1.66)	0.60
**Cumulative breastfeeding duration/month**^**c**^														
Q1: 1–< 39	64	24.3	88	33.2	1.00 (Ref)		15	18.3	1.00 (Ref)		20	13.9	1.00 (Ref)	
Q2: 39–< 62	59	22.4	68	25.7	0.99 (0.54, 1.80)	0.97	22	26.8	2.48 (0.97, 6.33)	0.058	40	27.8	**2.98 (1.33, 6.71)**	**0.008**
Q3: 62–< 96	71	27.0	53	20.0	0.66 (0.33, 1.30)	0.23	14	17.1	0.80 (0.27, 2.39)	0.69	40	27.8	1.80 (0.74, 4.38)	0.20
Q4: ≥ 96	69	26.2	56	21.1	0.99 (0.44, 2.20)	0.97	31	37.8	1.30 (0.40, 4.16)	0.66	44	30.6	1.87 (0.69, 5.07)	0.22
Trend‡					0.93 (0.71, 1.20)	0.56			0.97 (0.67, 1.41)	0.88			1.12 (0.82, 1.52)	0.47
**Mean breastfeeding duration per child/month**														
< 12	34	12.9	48	18.1	1.00 (Ref)		11	13.4	1.00 (Ref)		23	16.0	1.00 (Ref)	
12– 23	142	54.0	139	52.5	0.65 (0.35, 1.21)	0.18	49	59.8	1.24 (0.47, 3.29)	0.66	71	49.3	0.75 (0.36, 1.59)	0.46
≥ 24	87	33.1	78	29.4	0.64 (0.32, 1.27)	0.20	22	26.8	1.21 (0.42, 3.52)	0.73	50	34.7	1.09 (0.49, 2.43)	0.83
Trend‡					0.84 (0.60, 1.16)	0.28			1.07 (0.66, 1.72)	0.80			1.13 (0.76, 1.66)	0.55
**Age at first pregnancy and Number of children**														
Age 25+ years, 1–3 births	54	20.7	64	23.5	1.00 (Ref)		15	17.9	1.00 (Ref)		20	13.6	1.00 (Ref)	
Age < 25 years, 1–3 births	80	30.7	93	34.2	0.93 (0.52, 1.67)	0.81	19	22.6	0.82 (0.31, 2.15)	0.69	47	32.0	1.39 (0.65, 2.97)	0.40
Age 25+ years, 4+ births	15	5.7	18	6.6	0.77 (0.29, 2.05)	0.60	8	9.5	2.10 (0.58, 7.54)	0.26	7	4.8	1.12 (0.29, 4.27)	0.87
Age < 25 years, 4+ births	112	42.9	97	35.7	0.55 (0.29, 1.05)	0.068	42	50.0	1.31 (0.51, 3.39)	0.58	73	49.7	1.38 (0.62, 3.08)	0.43
Trend‡					**0.81 (0.66, 0.99)**	**0.041**			1.16 (0.86, 1.56)	0.33			1.07 (0.84, 1.37)	0.58
**Number of children and Cumulative breastfeeding duration**														
Nulliparous or ≤ 3 children and < 62 months	117	42.2	146	51.2	1.00 (Ref)		31	36.0	1.00 (Ref)		51	34.7	1.00 (Ref)	
≤ 3 children and ≥ 62 months	30	10.8	25	8.8	0.61 (0.30, 1.22)	0.16	6	7.0	0.41 (0.11, 1.56)	0.19	17	11.6	1.19 (0.53, 2.66)	0.68
≥ 4 children and < 62 months	20	7.2	30	10.5	0.89 (0.38, 2.07)	0.79	10	11.6	2.34 (0.78, 6.99)	0.13	12	8.2	1.31 (0.49, 3.53)	0.59
≥ 4 children and ≥ 62 months	110	39.7	84	29.5	**0.52 (0.30, 0.89)**	**0.02**	39	45.3	1.26 (0.57, 2.78)	0.56	67	45.6	0.96 (0.50, 1.84)	0.91
Trend‡					**0.81 (0.68, 0.97)**	**0.02**			1.11 (0.85, 1.44)	0.44			0.99 (0.80, 1.22)	0.91
**Menopausal status**^**a**^														
Premenopausal	144	50.3	174	59.4	1.00 (Ref)		44	50.0	1.00 (Ref)		68	44.7	1.00 (Ref)	
Postmenopausal	142	49.7	119	40.6	0.69 (0.32, 1.48)	0.33	44	50.0	0.69 (0.22, 2.10)	0.51	84	55.3	1.82 (0.78, 4.28)	0.17
**Age at menopause/year**														
Premenopausal	144	50.3	174	59.4	*N/A*		44	50.0	*N/A*		68	44.7	*N/A*	
< 50 years	58	22.5	58	20.9	1.00 (Ref)		28	33.7	1.00 (Ref)		44	32.1	1.00 (Ref)	
> 50 years	56	21.7	46	16.6	0.88 (0.45, 1.74)	0.72	11	13.3	**0.38 (0.15, 0.997)**	**0.049**	25	18.3	0.66 (0.31, 1.40)	0.28

*Seventeen cases were excluded from analyses because of their missing data for HER2 status. † Point estimates and 95% confidence intervals were from multivariable models, adjusting for the same series of covariates (except where noticed): age at diagnosis, BMI, age at menarche, age at first pregnancy, number of children, mean breastfeeding duration per child, age at menopause, family history of breast cancer in first-degree female relative, occupation, education level, and location of the facility. Estimates of numbers of children, cumulative and mean breastfeeding duration, and combined age at first pregnancy, and number of children were computed among parous women. ‡ Results were from the trend analysis using the categorical risk factor as a trend. ^a^ Multivariable modeling analysis without adjusting for age at menopause. ^b^Women who reported never pregnant, never gave birth, and had no child were grouped as "Nulliparous" in modeling analyses. ^c^ Multivariable modeling analysis without adjusting for mean breastfeeding duration per child. BMI, body mass index; CI, confidence interval; HER2, human epidermal growth factor receptor-2; OR, odds ratio; Q, quartile

We also conducted a number of sensitivity analyses to evaluate the impact of using grade to define subtypes when ki67 was missing and removing nulliparous women from analyses of age at first birth on our main conclusions. Overall, the results were similar to those from the original analyses (Supplementary Tables 7, 8, 9).

## Discussion

The etiology of early-onset breast cancers is particularly lacking across populations given their rarity. Studying African populations where risk factors differ and where onset is almost a decade earlier could provide new insights on breast cancer etiology given the etiologic and molecular subtype heterogeneity in diverse populations.

There is limited data from Africa where some of the breast cancer-associated risk/protective factors such as parity and breastfeeding have extremely different distributions. The overall risk factor distribution for BC patients in our study is similar to a large case-control study from Ghana [19], but is strikingly different from that of other populations including African Americans [20–22]. As an example, among BC patients in Ghana and Kenya, > 60% of women had ≥ 3 children, > 80% women had the first child before age 25 years, and > 90% women had breastfed with the average breastfeeding duration per child near two years. Whereas among African American BC patients in the African American Breast Cancer Epidemiology and Risk (AMBER) consortium, only 35% had ≥ 3 children and > 40% had never breastfed [21]. Similarly, the prevalence of obesity (BMI > 30 kg/m^2^, 41.7% in AMBER vs. 29.4% in Kenya) and early age at menarche (< 13 years, 52.3% in AMBER vs. 8.5% in Kenya) was much higher in AMBER [22, 23] than in Kenya. On the other hand, the frequency of ER-negative cancers (AMBER: 33.9%; Kenya: 30.5%) and TNBC (AMBER: 15.3%; Kenya: 18.6%) was similar in AMBER and Kenya, which is lower compared to BC patients in Ghana (ER−: 50%; TNBC: 28%).

Parity has been reported to have a dual effect on breast cancer risk; it is protective for ER+ women while increases risk for ER− women especially among younger women [24, 21]. Despite the heterogeneity in parity-related exposures, the differential effect of parity by ER has been consistently reported across different populations [25, 21, 19, 26]. Although we were not able to compare relative risks associated with parity in different molecular subtypes due to the case-only design, our results of higher parity in ER-negative than in ER-positive patients is consistent with results from previous case-control studies [19, 26]. In particular, taking advantage of the much higher parity among patients in Kenya, we observed that the association of parity with ER followed a dose-dependent manner, with the highest variation by ER observed among women with five or more children. Similarly, in a population where the vast majority of women had their first children before the age of 30 years, we found a similar association between younger age at first birth and ER-negative breast cancer consistent with previous studies [27, 26, 28], supporting increased parity as a risk factor for ER-negative breast cancers across multiple populations. We observed luminal B patients, both luminal B/high proliferative and luminal B/HER2+, had fewer children compared to luminal A patients. These results are in line with data from the Nurse’s Health Study reporting greater reduced risks associated with parity in luminal B than luminal A patients [25], suggesting that parity may have a stronger protective effect for luminal B as compared to luminal A patients. However, using data based on a Malaysian case-series, we found that luminal B patients were more likely to be parous and to have breastfed compared to luminal A patients [26]. These inconsistent results warrant further investigations especially in diverse populations.

Investigations of associations between breastfeeding and breast cancer risk by receptor status have resulted in inconsistent findings, with some showing a similar protective effect for all subtypes [29], and others showing a stronger protection against ER-negative especially TNBC [30]. In the Ghana study in which the frequency of ER-negative breast cancer especially TNBC was higher (28% vs 18% of tumors) than in the Kenya study, the increased risk of parity was offset by more extended breastfeeding, which was only seen among patients < 50 years of age in ER-negative but not in ER-positive patients, while in older women, extended breastfeeding showed an inverse association regardless of ER status yet a stronger association for ER-positive patients [19]. We did not observe significant differences of breastfeeding by ER or by intrinsic subtype, either in all women or by age. The inconsistent findings between different African populations with similar parity and breastfeeding characteristics highlight the complexity of subtype-specific risk associations and the importance of conducting large molecular epidemiologic studies in diverse African populations.

Obesity is a known risk factor for breast cancer in post-menopausal women but protective in premenopausal women [31]. Obesity can disrupt some biological pathways, resulting in insulin resistance, and synthesis of endogenous sex hormones [32, 33]. When we examined the association of obesity with molecular subtypes, we found that patients with HER2 enriched BC were less likely to have a high BMI. Although we cannot completely rule out the possibility of reverse causality due to weight loss associated with breast cancer, it is unlikely that the association we observed is entirely driven by reverse causation since BMI did not vary significantly by tumor stage in our study. Our findings are consistent with a Polish breast cancer case-control study, which found that in premenopausal women, HER2 expression was inversely associated with BMI adjusted for the 4 markers (adjusted *p*-trend = 0.01) [34]. In addition, the association was stronger among AKU patients, who were more likely to have early-stage disease as compared to patients from other hospitals. Our findings are similar to a study conducted in Malaysia, which showed that women with HER2-enriched and TNBC tumors were significantly less likely to be obese than those with the luminal A subtype [26]. Our results are also in line with the analysis based on African Americans in the AMBER consortium [22] and a pooled analysis of nine studies of the National Cancer Institute cohort consortium [27] showing that, among postmenopausal women, higher recent BMI was associated with increased risk of ER-positive cancer, but was either associated with decreased risk of ER-negative tumors in AMBER or was not associated with ER-negative BC in the NCI cohort consortium. Notably, the association with BMI observed in our study was mostly driven by HER2 status rather than by TNBC, which is more similar to the findings in the Malaysian study [26].

The strength of our study includes representation of BC cases from multiple hospitals in Kenya, well-annotated risk factor questionnaire and clinical data, and centralized high-quality biomarker assessment in a unique east African population.

This study was limited by the retrospective collection of risk factor data and possible reverse causation, as well as the case-only design, which prohibited us from estimating relative risks associated with each risk factor. Further, despite being the largest BC study of this type conducted in Kenya, the sample size was still relatively small to evaluate risk factors in rare tumor subtypes, especially in age-stratified analyses.

## Conclusion

In summary, our findings, based on data from an indigenous African population with unique risk factor profiles, add to the growing body of knowledge regarding the etiologic heterogeneity of breast cancer molecular subtypes among geographically diverse ethnic groups. Further investigations of genetic and environmental factors that modify breast cancer risk in African populations are recommended. Inclusion of diverse regional population groups from sub-Saharan Africa in global breast cancer studies may help provide a better understanding of the subtype-specific breast cancer risk etiology, which will be critical for the development of risk prediction models in African populations.

## Supplementary Information

**Supplementary Table 1..** Classifications of the five hospital groups

**Supplementary Table 2. **Associations between breast cancer risk factors and PR and HER2 status in Kenyan breast cancer patients (*N*=838)

**Supplementary Table 3. **Associations of key risk factors with ER status by hospitals (*N*=838)

**Supplementary Table 4. **Associations between BMI and HER2 status stratified by hospitals (*N*=821)

**Supplementary Table 5. **Associations between key risk factors and ER status by age at diagnosis (*N*=834)

**Supplementary Table 6. **Associations between breast cancer risk factors and tumor molecular subtypes in Kenyan breast cancer patients (*N*=776)

**Supplementary Table 7..** Associations of key risk factors and tumor subtypes without applying tumor grade to tumor subtype classification

**Supplementary Table 8.** Associations of key risk factors and tumor subtypes after excluding women missing for ki67

**Supplementary Table 9..** Associations between age at first pregnancy and ER status .in parous women

**Supplementary Figure 1..** Associations between BMI and HER2 status stratified by hospital groups

## Data Availability

The datasets used and/or analyzed during the current study are available from the corresponding author on reasonable request.

## Abbreviations

AKU: Aga Khan University
AMBER: African American Breast Cancer Epidemiology and Risk
ASR: Age-standardized rates
BC: Breast cancer
BMI: Body mass index
CA: Caucasian-American
CI: Confidence interval
CK: Cytokeratin
EGFR: Epidermal growth factor receptor
ER: Estrogen receptor
FISH: Fluorescence in situ hybridization
HDI: Human development index
HER2: Epidermal growth factor receptor 2
HREC: Health Sciences Research Ethics Committee
IHC: Immunohistochemistry
IQR: Interquartile range
NACOSTI: National Commission for Science Technology and Innovation
PGH: Provincial General Hospital
PR: Progesterone receptor
Q1: Quartile 1
Q2: Quartile 2
Q3: Quartile 3
Q4: Quartile 4
REC: Research Ethics Committees
RS: Relative survival
SAS: Statistical software
SD: Standard deviation
SSA: Sub-Saharan Africa
TBCCC: Tianjin Cohort of Breast Cancer Cases
TDLU: Terminal duct lobular unit
TN: Triple-negative
TNBC: Triple-negative breast cancer
UCT: University of Cape Town
