# Supplementary material for: Breast cancer risk factors in relation to molecular subtypes in breast cancer patients from Kenya
Source: Breast Cancer Res. 2021 Jun 26;23:68. doi: 10.1186/s13058-021-01446-3 (PMC8235821; doi:10.1186/s13058-021-01446-3)
Supplement: Supplementary file 1 — Supplementary Table 1.. Classifications of the five hospital groups [file 13058_2021_1446_MOESM1_ESM.docx]

**Supplementary Table 1.** **Classifications of five hospital groups**

| **Hospital groups** | **N** | **%** |
| --- | --- | --- |
| Aga Khan University (AKU) hospital (private) | 240 | 28.6 |
| AKU, Kisumu, Kenya | 57 | 6.8 |
| AKU, Mombasa, Kenya | 16 | 1.9 |
| AKU, Nairobi | 277 | 33.1 |
|  |  |  |
| Kijabe Mission Hospital, AIC (faith-based) | 105 | 12.5 |
|  |  |  |
| Nyeri Provincial General Hospital (public) | 151 | 18.0 |
|  |  |  |
| St. Mary's Mission Hospital, Nairobi (faith-based) | 122 | 14.6 |
|  |  |  |
| Other | 582 | 69.5 |
| Garissa Provincial General Hospital | 3 | 0.4 |
| Jaramogi Oginga Odinga Teaching and Referral  Hospital | 46 | 5.5 |
| Kenyatta National Hospital, Nairobi | 85 | 10.1 |
| Kisii General Hospital | 4 | 0.5 |
| Moi Teaching and Referral Hospital, Eldoret | 5 | 0.6 |
| Tenwek Mission Hospital | 8 | 1.0 |
