# Supplementary material for: Breast cancer risk factors in relation to molecular subtypes in breast cancer patients from Kenya
Source: Breast Cancer Res. 2021 Jun 26;23:68. doi: 10.1186/s13058-021-01446-3 (PMC8235821; doi:10.1186/s13058-021-01446-3)
Supplement: Supplementary file 2 — Supplementary Table 2. Associations between breast cancer risk factors and PR and HER2 status in Kenyan breast cancer patients (N=838) [file 13058_2021_1446_MOESM2_ESM.docx]

**Supplementary Table 2. Associations between breast cancer risk factors and PR and HER2 status in Kenyan breast cancer patients (N=838)**

|  | **PR⁺ N=498** | | **PR⁻ N=340** | | **PR⁻ vs. PR⁺** | |  | **HER2⁻ N=596** | | **HER2⁺ N=225** | | **HER2+ vs. HER2⁻** | |
| --- | --- | --- | --- | --- | --- | --- | --- | --- | --- | --- | --- | --- | --- |
|  | **N** | **%** | **N** | **%** | **OR (95% CI)†** | ***P†*** |  | **N** | **%** | **N** | **%** | **OR (95% CI)†** | ***P†*** |
| **Age at diagnosis/year** |  |  |  |  |  |  |  |  |  |  |  |  |  |
| 20-39 | 132 | 26.7 | 77 | 22.6 | 1.00 (Ref) |  |  | 139 | 23.5 | 68 | 30.2 | 1.00 (Ref) |  |
| 40-49 | 144 | 29.1 | 98 | 28.8 | 1.20 (0.75, 1.93) | 0.45 |  | 176 | 29.7 | 60 | 26.7 | 0.79 (0.47, 1.33) | 0.38 |
| 50-59 | 117 | 23.7 | 94 | 27.6 | 1.15 (0.52, 2.58) | 0.73 |  | 145 | 24.5 | 63 | 28.0 | 1.87 (0.76, 4.64) | 0.18 |
| ≥60 | 101 | 20.4 | 71 | 20.9 | 0.84 (0.32, 2.21) | 0.73 |  | 132 | 22.3 | 34 | 15.1 | 1.42 (0.46, 4.38) | 0.54 |
| Trend‡ |  |  |  |  | 0.98 (0.72, 1.34) | 0.90 |  |  |  |  |  | 1.02 (0.72, 1.45) | 0.90 |
| **BMI/ kg/m^2^** |  |  |  |  |  |  |  |  |  |  |  |  |  |
| Normal (<25.0) | 116 | 29.4 | 94 | 34.1 | 1.00 (Ref) |  |  | 139 | 29.1 | 64 | 36.0 | 1.00 (Ref) |  |
| Overweight (25.0 - 29.9) | 157 | 39.7 | 107 | 38.8 | 0.80 (0.53, 1.21) | 0.29 |  | 187 | 39.2 | 70 | 39.3 | 0.77 (0.49, 1.21) | 0.25 |
| Obese (≥30.0) | 122 | 30.9 | 75 | 27.2 | 0.68 (0.43, 1.09) | 0.11 |  | 151 | 31.7 | 44 | 24.7 | **0.58 (0.34, 0.97)** | **0.038** |
| Trend‡ |  |  |  |  | 0.83 (0.66, 1.05) | 0.11 |  |  |  |  |  | **0.76 (0.59, 0.98)** | **0.038** |
| **Premenopausal: BMIᵃ** |  |  |  |  |  |  |  |  |  |  |  |  |  |
| Normal (<25.0) | 79 | 34.8 | 51 | 38.35 | 1.00 (Ref) |  |  | 86 | 34.96 | 40 | 37.38 | 1.00 (Ref) |  |
| Overweight (25.0 - 29.9) | 88 | 38.8 | 44 | 33.08 | 0.75 (0.42, 1.31) | 0.31 |  | 90 | 36.59 | 39 | 36.45 | 0.96 (0.53, 1.72) | 0.88 |
| Obese (≥30.0) | 60 | 26.4 | 38 | 28.57 | 0.82 (0.44, 1.51) | 0.52 |  | 70 | 28.46 | 28 | 26.17 | 0.86 (0.45, 1.66) | 0.66 |
| Trend‡ |  |  |  |  | 0.90 (0.66, 1.22) | 0.49 |  |  |  |  |  | 0.93 (0.67, 1.29) | 0.66 |
| **Postmenopausal: BMIᵃ** |  |  |  |  |  |  |  |  |  |  |  |  |  |
| Normal (<25.0) | 37 | 22.2 | 43 | 30.07 | 1.00 (Ref) |  |  | 53 | 22.94 | 24 | 34.29 | 1.00 (Ref) |  |
| Overweight (25.0 - 29.9) | 69 | 41.3 | 63 | 4.06 | 0.92 (0.48, 1.75) | 0.79 |  | 97 | 41.99 | 31 | 44.29 | 0.48 (0.23, 1.02) | 0.056 |
| Obese (≥30.0) | 61 | 36.5 | 37 | 25.87 | 0.65 (0.32, 1.31) | 0.23 |  | 81 | 35.06 | 15 | 21.43 | **0.26 (0.10, 0.62)** | **0.0027** |
| Trend‡ |  |  |  |  | 0.81 (0.57, 1.15) | 0.24 |  |  |  |  |  | **0.50 (0.32, 0.79)** | **0.0026** |
| **Age at menarche/year** |  |  |  |  |  |  |  |  |  |  |  |  |  |
| ≤13 (9-13) | 118 | 24.9 | 80 | 24.4 | 1.00 (Ref) |  |  | 140 | 24.8 | 56 | 25.5 | 1.00 (Ref) |  |
| 14 | 128 | 27.0 | 82 | 25.0 | 1.19 (0.74, 1.90) | 0.48 |  | 139 | 24.6 | 61 | 27.7 | 1.34 (0.80, 2.26) | 0.27 |
| ≥15 (15-20) | 228 | 48.1 | 166 | 50.6 | 1.48 (0.97, 2.26) | 0.07 |  | 286 | 50.6 | 103 | 46.8 | 0.98 (0.61, 1.56) | 0.92 |
| Trend‡ |  |  |  |  | 1.22 (0.99, 1.50) | 0.06 |  |  |  |  |  | 0.96 (0.77, 1.21) | 0.76 |
| **Age at first pregnancy/year** |  |  |  |  |  |  |  |  |  |  |  |  |  |
| <20 | 114 | 23.5 | 102 | 30.4 | 1.00 (Ref) |  |  | 151 | 26.0 | 61 | 27.2 | 1.00 (Ref) |  |
| 20-24 | 219 | 45.2 | 142 | 42.4 | 0.84 (0.53, 1.33) | 0.46 |  | 270 | 46.6 | 83 | 37.1 | 0.70 (0.41, 1.19) | 0.19 |
| 25-29 | 94 | 19.4 | 56 | 16.7 | 1.02 (0.56, 1.86) | 0.95 |  | 101 | 17.4 | 47 | 21.0 | 1.02 (0.53, 1.98) | 0.94 |
| Nulliparousᵇ or age ≥30 | 58 | 12.0 | 35 | 10.4 | 1.04 (0.46, 2.37) | 0.92 |  | 58 | 10.0 | 33 | 14.7 | 1.17 (0.49, 2.83) | 0.72 |
| Trend‡ |  |  |  |  | 1.03 (0.81, 1.31) | 0.83 |  |  |  |  |  | 1.08 (0.82, 1.41) | 0.59 |
| **Parity** |  |  |  |  |  |  |  |  |  |  |  |  |  |
| Nulliparousᵇ | 27 | 5.4 | 17 | 5.0 | 1.02 (0.35, 3.02) | 0.97 |  | 25 | 4.2 | 16 | 7.1 | 1.02 (0.32, 3.18) | 0.98 |
| Parous | 471 | 94.6 | 323 | 95.0 | 1.00 (Ref) |  |  | 571 | 95.8 | 209 | 92.9 | 1.00 (Ref) |  |
| **Number of children** |  |  |  |  |  |  |  |  |  |  |  |  |  |
| 1 or 2 | 150 | 31.8 | 76 | 23.5 | 1.00 (Ref) |  |  | 156 | 27.3 | 68 | 32.5 | 1.00 (Ref) |  |
| 3 or 4 | 186 | 39.5 | 121 | 37.5 | 1.35 (0.86, 2.14) | 0.20 |  | 227 | 39.8 | 72 | 34.4 | 0.66 (0.40, 1.10) | 0.11 |
| ≥ 5 | 135 | 28.7 | 126 | 39.0 | 1.64 (0.94, 2.86) | 0.08 |  | 188 | 32.9 | 69 | 33.0 | 0.91 (0.49, 1.70) | 0.77 |
| Trend‡ |  |  |  |  | 1.28 (0.97, 1.69) | 0.08 |  |  |  |  |  | 0.94 (0.68, 1.29) | 0.69 |
| **Cumulative breastfeeding**  **duration/month^c^** |  |  |  |  |  |  |  |  |  |  |  |  |  |
| Q1: 1 - <39 | 133 | 29.2 | 58 | 18.5 | 1.00 (Ref) |  |  | 133 | 24.1 | 54 | 26.6 | 1.00 (Ref) |  |
| Q2: 39 - <62 | 106 | 23.3 | 86 | 27.5 | **1.95 (1.16, 3.29)** | **0.012** |  | 137 | 24.9 | 52 | 25.6 | 1.13 (0.65, 1.98) | 0.66 |
| Q3: 62 - <96 | 108 | 23.7 | 74 | 23.6 | 1.36 (0.74, 2.49) | 0.32 |  | 135 | 24.5 | 43 | 21.2 | 0.87 (0.44, 1.70) | 0.68 |
| Q4: ≥96 | 108 | 23.7 | 95 | 30.4 | 1.43 (0.72, 2.81) | 0.31 |  | 146 | 26.5 | 54 | 26.6 | 0.98 (0.46, 2.12) | 0.96 |
| Trend‡ |  |  |  |  | 1.09 (0.88, 1.36) | 0.44 |  |  |  |  |  | 0.97 (0.76, 1.25) | 0.83 |
| **Mean breastfeeding**  **duration per child/month** |  |  |  |  |  |  |  |  |  |  |  |  |  |
|  |  |  |  |  |  |  |  |  |  |  |  |  |  |
| <12 | 70 | 15.4 | 50 | 16.0 | 1.00 (Ref) |  |  | 84 | 15.2 | 32 | 15.8 | 1.00 (Ref) |  |
| 12- 23 | 243 | 53.4 | 164 | 52.4 | 1.00 (0.59, 1.69) | 0.99 |  | 285 | 51.7 | 116 | 57.1 | 1.00 (0.56, 1.79) | 1.00 |
| ≥ 24 | 142 | 31.2 | 99 | 31.6 | 1.31 (0.74, 2.32) | 0.35 |  | 182 | 33.0 | 55 | 27.1 | 0.71 (0.37, 1.36) | 0.31 |
| Trend‡ |  |  |  |  | 1.18 (0.89, 1.55) | 0.24 |  |  |  |  |  | 0.82 (0.60, 1.12) | 0.21 |
| **Age at first pregnancy &**  **Number of children** |  |  |  |  |  |  |  |  |  |  |  |  |  |
| Age 25+ yr, 1-3 births | 103 | 22.5 | 51 | 16.0 | 1.00 (Ref) |  |  | 107 | 19.3 | 46 | 22.0 | 1.00 (Ref) |  |
| Age <25 yr, 1-3 births | 146 | 31.9 | 98 | 30.7 | 1.17 (0.70, 1.97) | 0.55 |  | 180 | 32.4 | 59 | 28.2 | 0.74 (0.42, 1.30) | 0.29 |
| Age 25+ yr, 4+ births | 24 | 5.2 | 25 | 7.8 | **2.30 (1.01, 5.25)** | **0.048** |  | 29 | 5.2 | 19 | 9.1 | 1.65 (0.69, 3.92) | 0.26 |
| Age <25 yr, 4+ births | 185 | 40.4 | 145 | 45.5 | 1.17 (0.67, 2.03) | 0.58 |  | 239 | 43.1 | 85 | 40.7 | 0.88 (0.48, 1.61) | 0.69 |
| Trend‡ |  |  |  |  | 1.05 (0.89, 1.25) | 0.07 |  |  |  |  |  | 1.19 (0.99, 1.43) | 0.06 |
| **Number of children & Cumulative breastfeeding duration** |  |  |  |  |  |  |  |  |  |  |  |  |  |
| Nulliparous or ≤3 children  & <62 months | 224 | 46.5 | 130 | 39.4 | 1.00 (Ref) |  |  | 246 | 42.7 | 99 | 45.2 | 1.00 (Ref) |  |
| ≤3 children & ≥62 months | 47 | 9.8 | 32 | 9.7 | 1.19 (0.66, 2.17) | 0.57 |  | 60 | 10.4 | 18 | 8.2 | 0.66 (0.32, 1.34) | 0.25 |
| ≥4 children & <62 months | 42 | 8.7 | 31 | 9.4 | 1.29 (0.65, 2.53) | 0.47 |  | 49 | 8.5 | 23 | 10.5 | 1.80 (0.86, 3.77) | 0.12 |
| ≥4 children & ≥62 months | 169 | 35.1 | 137 | 41.5 | 1.16 (0.74, 1.83) | 0.51 |  | 221 | 38.4 | 79 | 36.1 | 1.11 (0.66, 1.85) | 0.70 |
| Trend‡ |  |  |  |  | 1.05 (0.91, 1.22) | 0.50 |  |  |  |  |  | 1.05 (0.89, 1.25) | 0.58 |
| **Menopausal status^a^** |  |  |  |  |  |  |  |  |  |  |  |  |  |
| Premenopausal | 276 | 55.5 | 162 | 47.8 | 1.00 (Ref) |  |  | 303 | 50.9 | 127 | 56.7 | 1.00 (Ref) |  |
| Postmenopausal | 221 | 44.5 | 177 | 52.2 | 1.68 (0.89, 3.16) | 0.11 |  | 292 | 49.1 | 97 | 43.3 | 0.49 (0.23, 1.06) | 0.07 |
| **Age at menopause/year^d^** |  |  |  |  |  |  |  |  |  |  |  |  |  |
| < 50 | 95 | 20.7 | 96 | 30.7 | 1.00 (Ref) |  |  | 129 | 23.8 | 59 | 27.4 | 1.00 (Ref) |  |
| > 50 | 87 | 19.0 | 55 | 17.6 | 0.73 (0.41, 1.31) | 0.30 |  | 109 | 20.1 | 29 | 13.5 | 0.66 (0.32, 1.38) | 0.27 |
| **Family history of breast cancer in first degree female relatives** |  |  |  |  |  |  |  |  |  |  |  |  |  |
|  |  |  |  |  |  |  |  |  |  |  |  |  |  |
| No | 459 | 92.2 | 314 | 92.4 | 1.00 (Ref) |  |  | 542 | 90.9 | 216 | 96.0 | 1.00 (Ref) |  |
| Yes | 39 | 7.8 | 26 | 7.6 | 0.94 (0.50, 1.78) | 0.85 |  | 54 | 9.1 | 9 | 4.0 | **0.39 (0.16, 0.93)** | **0.033** |
| **Alcohol Use** |  |  |  |  |  |  |  |  |  |  |  |  |  |
| No | 443 | 89.0 | 317 | 93.2 | 1.00 (Ref) |  |  | 548 | 91.9 | 197 | 87.6 | 1.00 (Ref) |  |
| Yes | 55 | 11.0 | 23 | 6.8 | 0.69 (0.37, 1.28) | 0.24 |  | 48 | 8.1 | 28 | 12.4 | **2.49 (1.35, 4.61)** | **0.0036** |

† Point estimates and 95% confidence intervals were from multivariable models, adjusting for the same series of covariates (except where noticed): age at diagnosis, BMI, age at menarche, age at first pregnancy, number of children, mean breastfeeding duration per child, age at menopause, family history of breast cancer in first degree female relative, occupation, education level, and location of facility. Estimates of numbers of children, cumulative and averaged breastfeeding duration, and combined age at first pregnancy and number of children were computed among parous women. ‡ Results were from the trend analysis treating the categorical risk factor as a semi-continuous variable. ᵃ Multivariable modeling analysis without adjusting for age at menopause. ᵇ Women who reported never pregnant, never gave birth, and had no child were grouped as "Nulliparous" in modeling analyses. ^c^ Multivariable modeling analysis without adjusting for mean breastfeeding duration per child. ^d^ Multivariable Modeling analysis was restricted to postmenopausal women. BMI, body mass index; CI, confidence interval; HER2, human epidermal growth factor receptor-2; PR, progesterone receptor; OR, odds ratio; Q, quartile.
