# Supplementary material for: Breast cancer risk factors in relation to molecular subtypes in breast cancer patients from Kenya
Source: Breast Cancer Res. 2021 Jun 26;23:68. doi: 10.1186/s13058-021-01446-3 (PMC8235821; doi:10.1186/s13058-021-01446-3)
Supplement: Supplementary file 3 — Supplementary Table 3. Associations of key risk factors with ER status by hospitals (N=838) [file 13058_2021_1446_MOESM3_ESM.docx]

Supplementary Table 3. Associations of key risk factors with ER status by hospitals (N=838)

|  | **Hospitals** | | | | | | | | | | | | | | | | | | | | | | | | | | | | | | | | | |
| --- | --- | --- | --- | --- | --- | --- | --- | --- | --- | --- | --- | --- | --- | --- | --- | --- | --- | --- | --- | --- | --- | --- | --- | --- | --- | --- | --- | --- | --- | --- | --- | --- | --- | --- |
|  | **AKU (n=350, 42%)** | | | | | |  | **Kijabe (n=105, 13%)** | | | | | |  | **Nyeri (n=110, 13%)** | | | | | |  | **St Mary's (n=122, 15%)** | | | | | |  | **Others (n=151, 18%)** | | | | | |
|  | **ER+** | | **ER-** | | **ER- vs. ER+** | |  | **ER+** | | **ER-** | | **ER- vs. ER+** | |  | **ER+** | | **ER-** | | **ER- vs. ER+** | |  | **ER+** | | **ER-** | | **ER- vs. ER+** | |  | **ER+** | | **ER-** | | **ER- vs. ER+** | |
| **Key risk factors** | **N** | **%** | **N** | **%** | **OR (95% CI)†** | **P†** |  | **N** | % | **N** | % | **OR (95% CI)†** | **P†** |  | **N** | % | **N** | % | **OR (95% CI)†** | **P†** |  | **N** | % | **N** | % | **OR (95% CI)†** | **P†** |  | **N** | % | **N** | % | **OR (95% CI)†** | **P†** |
| **BMI** |  |  |  |  |  |  |  |  |  |  |  |  |  |  |  |  |  |  |  |  |  |  |  |  |  |  |  |  |  |  |  |  |  |  |
| Normal (<25.0) | 42 | 18.9 | 25 | 26.9 | 1.00 (Ref) |  |  | 27 | 40.9 | 8 | 38.1 | 1.00 (Ref) |  |  | 12 | 36.4 | 13 | 43.3 | 1.00 (Ref) |  |  | 28 | 43.1 | 8 | 33.3 | 1.00 (Ref) |  |  | 27 | 38.0 | 20 | 43.5 | 1.00 (Ref) |  |
| Overweight (25.0 - 29.9) | 96 | 43.2 | 30 | 32.3 | **0.47 (0.25, 0.91)** | **0.026** |  | 25 | 37.9 | 8 | 38.1 | 1.20 (0.38, 3.82) | 0.75 |  | 15 | 45.5 | 13 | 43.3 | 0.86 (0.27, 2.68) | 0.79 |  | 25 | 38.5 | 12 | 50.0 | 1.74 (0.61, 5.00) | 0.30 |  | 24 | 33.8 | 16 | 34.8 | 0.91 (0.37, 2.24) | 0.84 |
| Obese (≥30.0) | 84 | 37.8 | 38 | 40.9 | 0.67 (0.35, 1.27) | 0.22 |  | 14 | 21.2 | 5 | 23.8 | 1.07 (0.28, 4.02) | 0.92 |  | 6 | 18.2 | 4 | 13.3 | 0.71 (0.15, 3.40) | 0.67 |  | 12 | 18.5 | 4 | 16.7 | 1.16 (0.29, 4.68) | 0.84 |  | 20 | 28.2 | 10 | 21.7 | 0.68 (0.26, 1.82) | 0.44 |
| Trend‡ |  |  |  |  | 0.87 (0.63, 1.20) | 0.40 |  |  |  |  |  | 1.05 (0.55, 2.00) | 0.88 |  |  |  |  |  | 0.85 (0.40, 1.78) | 0.66 |  |  |  |  |  | 1.16 (0.61, 2.21) | 0.65 |  |  |  |  |  | 0.83 (0.51, 1.35) | 0.45 |
| **Age at menarche/year** |  |  |  |  |  |  |  |  |  |  |  |  |  |  |  |  |  |  |  |  |  |  |  |  |  |  |  |  |  |  |  |  |  |  |
| ≤14 (9-14) | 125 | 50.2 | 55 | 54.5 | 1.00 (Ref) |  |  | 29 | 42.0 | 12 | 57.1 | 1.00 (Ref) |  |  | 27 | 45.0 | 19 | 45.2 | 1.00 (Ref) |  |  | 52 | 59.1 | 16 | 50.0 | 1.00 (Ref) |  |  | 47 | 52.2 | 26 | 52.0 | 1.00 (Ref) |  |
| ≥15 (15-20) | 124 | 49.8 | 46 | 45.5 | 0.90 (0.55, 1.49) | 0.69 |  | 40 | 58.0 | 9 | 42.9 | 0.47 (0.15, 1.50) | 0.21 |  | 33 | 55.0 | 23 | 54.8 | 2.68 (0.75, 9.59) | 0.13 |  | 36 | 40.9 | 16 | 50.0 | **2.96 (1.08, 8.12)** | **0.036** |  | 43 | 47.8 | 24 | 48.0 | 1.02 (0.47, 2.22) | 0.96 |
| **Age at first pregnancy/year** |  |  |  |  |  |  |  |  |  |  |  |  |  |  |  |  |  |  |  |  |  |  |  |  |  |  |  |  |  |  |  |  |  |  |
| <25 | 138 | 55.4 | 66 | 65.3 | 1.00 (Ref) |  |  | 53 | 67.1 | 17 | 73.9 | 1.00 (Ref) |  |  | 50 | 80.6 | 38 | 84.4 | 1.00 (Ref) |  |  | 64 | 79.0 | 26 | 86.7 | 1.00 (Ref) |  |  | 78 | 80.4 | 47 | 88.7 | 1.00 (Ref) |  |
| ≥25-29 | 67 | 26.9 | 23 | 22.8 | 0.60 (0.33, 1.08) | 0.09 |  | 19 | 24.1 | 2 | 8.7 | 0.54 (0.11, 2.80) | 0.46 |  | 8 | 12.9 | 6 | 13.3 | 5.51 (0.50, 60.64) | 0.16 |  | 13 | 16.0 | 1 | 3.3 | 0.32 (0.04, 2.80) | 0.30 |  | 8 | 8.2 | 3 | 5.7 | 0.87 (0.19, 3.90) | 0.85 |
| Nulliparousᵃ or ≥30 | 44 | 17.7 | 12 | 11.9 | 0.53 (0.25, 1.13) | 0.10 |  | 7 | 8.9 | 4 | 17.4 | 1.57 (0.33, 7.53) | 0.57 |  | 4 | 6.5 | 1 | 2.2 | 1.43 (0.07, 28.47) | 0.82 |  | 4 | 4.9 | 3 | 10.0 | 2.75 (0.46, 16.55) | 0.27 |  | 11 | 11.3 | 3 | 5.7 | 0.42 (0.11, 1.63) | 0.21 |
| Trend‡ |  |  |  |  | **0.67 (0.49, 0.99)** | **0.043** |  |  |  |  |  | 1.08 (0.50, 2.33) | 0.84 |  |  |  |  |  | 1.97 (0.54, 7.18) | 0.31 |  |  |  |  |  | 1.23 (0.54, 2.77) | 0.63 |  |  |  |  |  | 0.68 (0.36, 1.26) | 0.22 |
| **Parity** |  |  |  |  |  |  |  |  |  |  |  |  |  |  |  |  |  |  |  |  |  |  |  |  |  |  |  |  |  |  |  |  |  |  |
| Nulliparousᵃ | 23 | 9.2 | 3 | 3.0 | *N* | |  | 4 | 5.0 | 2 | 8.0 | *N* | |  | 2 | 3.1 | 0 | 0.0 | *N* | |  | 1 | 1.1 | 1 | 3.1 | *N* | |  | 7 | 7.1 | 1 | 1.9 | *N* | |
| Parous | 226 | 90.8 | 98 | 97.0 | 1.00 (Ref) |  |  | 76 | 95.0 | 23 | 92.0 | 1.00 (Ref) |  |  | 63 | 96.9 | 45 | 100.0 | 1.00 (Ref) |  |  | 89 | 98.9 | 31 | 96.9 | 1.00 (Ref) |  |  | 91 | 92.9 | 52 | 98.1 | 1.00 (Ref) |  |
| **Number of children** |  |  |  |  |  |  |  |  |  |  |  |  |  |  |  |  |  |  |  |  |  |  |  |  |  |  |  |  |  |  |  |  |  |  |
| 1-3 | 145 | 64.2 | 54 | 55.1 | 1.00 (Ref) |  |  | 38 | 50.0 | 6 | 26.1 | 1.00 (Ref) |  |  | 30 | 47.6 | 23 | 51.1 | 1.00 (Ref) |  |  | 41 | 46.1 | 9 | 29.0 | 1.00 (Ref) |  |  | 39 | 42.9 | 16 | 30.8 | 1.00 (Ref) |  |
| 4 or 5 | 81 | 35.8 | 44 | 44.9 | 1.55 (0.90, 2.67) | 0.11 |  | 38 | 50.0 | 17 | 73.9 | 3.30 (0.93, 11.76) | 0.07 |  | 33 | 52.4 | 22 | 48.9 | 0.82 (0.28, 2.43) | 0.72 |  | 48 | 53.9 | 22 | 71.0 | 2.88 (0.86, 9.64) | 0.09 |  | 52 | 57.1 | 36 | 69.2 | 1.59 (0.69, 3.67) | 0.28 |
| **Cumulative breastfeeding**  **duration /month** |  |  |  |  |  |  |  |  |  |  |  |  |  |  |  |  |  |  |  |  |  |  |  |  |  |  |  |  |  |  |  |  |  |  |
| T1: 1 - <48 | 81 | 36.3 | 22 | 22.7 | 1.00 (Ref) |  |  | 32 | 43.2 | 4 | 20.0 | 1.00 (Ref) |  |  | 16 | 26.2 | 13 | 28.9 | 1.00 (Ref) |  |  | 31 | 37.8 | 6 | 19.4 | 1.00 (Ref) |  |  | 27 | 31.0 | 8 | 16.7 | 1.00 (Ref) |  |
| T2: 48 - <81 | 95 | 42.6 | 47 | 48.5 | 1.77 (0.95, 3.31) | 0.07 |  | 26 | 35.1 | 6 | 30.0 | 1.66 (0.34, 8.00) | 0.53 |  | 20 | 32.8 | 16 | 35.6 | 1.32 (0.34, 5.18) | 0.69 |  | 19 | 23.2 | 7 | 22.6 | 1.80 (0.43, 7.63) | 0.42 |  | 25 | 28.7 | 12 | 25.0 | 1.35 (0.41, 4.40) | 0.62 |
| T3: ≥81 | 47 | 21.1 | 28 | 28.9 | 2.00 (0.96, 4.18) | 0.06 |  | 16 | 21.6 | 10 | 50.0 | 4.54 (0.97, 21.21) | 0.055 |  | 25 | 41.0 | 16 | 35.6 | 1.17 (0.28, 4.93) | 0.83 |  | 32 | 39.0 | 18 | 58.1 | 2.60 (0.73, 9.35) | 0.14 |  | 35 | 40.2 | 28 | 58.3 | 2.93 (0.97, 8.83) | 0.056 |
| Trend‡ |  |  |  |  | 1.42 (0.99, 2.05) | 0.056 |  |  |  |  |  | **2.19 (1.00, 4.78)** | **0.049** |  |  |  |  |  | 1.08 (0.53, 2.21) | 0.84 |  |  |  |  |  | 1.60 (0.85, 3.00) | 0.14 |  |  |  |  |  | **1.76 (1.01, 3.04)** | **0.044** |
| **Mean breastfeeding**  **duration per child/month** |  |  |  |  |  |  |  |  |  |  |  |  |  |  |  |  |  |  |  |  |  |  |  |  |  |  |  |  |  |  |  |  |  |  |
|  |  |  |  |  |  |  |  |  |  |  |  |  |  |  |  |  |  |  |  |  |  |  |  |  |  |  |  |  |  |  |  |  |  |  |
| <12 | 39 | 17.5 | 14 | 14.4 | 1.00 (Ref) |  |  | 19 | 25.7 | 4 | 20.0 | 1.00 (Ref) |  |  | 3 | 4.9 | 5 | 11.1 | 1.00 (Ref) |  |  | 12 | 14.6 | 6 | 19.4 | 1.00 (Ref) |  |  | 11 | 12.6 | 7 | 14.6 | 1.00 (Ref) |  |
| 12-23 | 103 | 46.2 | 50 | 51.5 | 1.32 (0.63, 2.78) | 0.46 |  | 36 | 48.6 | 10 | 50.0 | 0.93 (0.23, 3.77) | 0.92 |  | 42 | 68.9 | 29 | 64.4 | 1.18 (0.19, 7.39) | 0.86 |  | 48 | 58.5 | 16 | 51.6 | 0.55 (0.15, 2.07) | 0.38 |  | 48 | 55.2 | 25 | 52.1 | 1.03 (0.29, 3.66) | 0.97 |
| ≥24 | 81 | 36.3 | 33 | 34.0 | 1.27 (0.58, 2.79) | 0.55 |  | 19 | 25.7 | 6 | 30.0 | 0.86 (0.17, 4.27) | 0.86 |  | 16 | 26.2 | 11 | 24.4 | 1.93 (0.22, 17.02) | 0.55 |  | 22 | 26.8 | 9 | 29.0 | 0.56 (0.11, 2.91) | 0.49 |  | 29 | 32.2 | 16 | 33.3 | 1.27 (0.34, 4.79) | 0.72 |
| Trend‡ |  |  |  |  | 1.31 (0.97, 1.77) | 0.08 |  |  |  |  |  | 0.86 (0.47, 1.59) | 0.63 |  |  |  |  |  | 1.60 (0.60, 4.27) | 0.35 |  |  |  |  |  | 0.63 (0.29, 1.36) | 0.24 |  |  |  |  |  | 1.34 (0.80, 2.25) | 0.26 |
| **Age at first pregnancy &**  **Number of children** |  |  |  |  |  |  |  |  |  |  |  |  |  |  |  |  |  |  |  |  |  |  |  |  |  |  |  |  |  |  |  |  |  |  |
| Age 25+ yr, 1-3 births | 74 | 32.7 | 24 | 24.5 | 1.00 (Ref) |  |  | 14 | 18.7 | 2 | 9.1 | 1.00 (Ref) |  |  | 8 | 13.3 | 5 | 11.1 | 1.00 (Ref) |  |  | 12 | 15.0 | 1 | 3.4 | 1.00 (Ref) |  |  | 9 | 10.0 | 5 | 9.6 | 1.00 (Ref) |  |
| Age <25 yr, 1-3 births | 71 | 31.4 | 30 | 30.6 | 1.50 (0.77, 2.96) | 0.24 |  | 23 | 30.7 | 4 | 18.2 | 0.46 (0.06, 3.86) | 0.48 |  | 20 | 33.3 | 18 | 40.0 | 0.27 (0.02, 3.05) | 0.29 |  | 29 | 36.3 | 8 | 27.6 | 2.38 (0.24, 23.83) | 0.46 |  | 30 | 33.3 | 11 | 21.2 | 0.63 (0.15, 2.56) | 0.52 |
| Age 25+ yr, 4+ births | 15 | 6.6 | 8 | 8.2 | 1.78 (0.65, 4.92) | 0.27 |  | 9 | 12.0 | 3 | 13.6 | 1.35 (0.15, 12.46) | 0.79 |  | 2 | 3.3 | 2 | 4.4 | *N* |  |  | 4 | 5.0 | 2 | 6.9 | 12.02 (0.64, 226.73) | 0.10 |  | 4 | 4.4 | 0 | 0 | *N* | |
| Age <25 yr, 4+ births | 66 | 29.2 | 36 | 36.7 | 1.93 (0.99, 3.80) | 0.06 |  | 29 | 38.7 | 13 | 59.1 | 2.17 (0.37, 12.56) | 0.39 |  | 30 | 50.0 | 20 | 44.4 | 0.23 (0.02, 2.59) | 0.23 |  | 35 | 43.8 | 18 | 62.1 | 5.18 (0.55, 48.72) | 0.15 |  | 47 | 52.2 | 36 | 69.2 | 1.22 (0.35, 4.26) | 0.76 |
| Trend‡ |  |  |  |  | 1.23 (0.99, 1.53) | 0.06 |  |  |  |  |  | 1.58 (0.91, 2.74) | 0.10 |  |  |  |  |  | 0.79 (0.48, 1.31) | 0.99 |  |  |  |  |  | 1.55 (0.92, 2.61) | 0.10 |  |  |  |  |  | 1.21 (0.85, 1.73) | 0.29 |
| **Number of children & Cumulative breastfeeding duration** |  |  |  |  |  |  |  |  |  |  |  |  |  |  |  |  |  |  |  |  |  |  |  |  |  |  |  |  |  |  |  |  |  |  |
| Nulliparous or ≤3 children  & <62 months | 134 | 54.5 | 45 | 45.0 | 1.00 (Ref) |  |  | 37 | 47.4 | 7 | 31.8 | 1.00 (Ref) |  |  | 23 | 36.5 | 20 | 44.4 | 1.00 (Ref) |  |  | 36 | 43.4 | 8 | 25.0 | 1.00 (Ref) |  |  | 35 | 37.2 | 9 | 18.4 | 1.00 (Ref) |  |
| ≤3 children & ≥62 months | 31 | 12.6 | 12 | 12.0 | 1.04 (0.45, 2.39) | 0.93 |  | 4 | 5.1 | 1 | 4.5 | 1.63 (0.13, 20.05) | 0.70 |  | 7 | 11.1 | 3 | 6.7 | 0.59 (0.10, 3.63) | 0.57 |  | 6 | 7.2 | 2 | 6.3 | 1.26 (0.11, 14.74) | 0.85 |  | 8 | 8.5 | 5 | 10.2 | 1.65 (0.37, 7.41) | 0.51 |
| ≥4 children & <62 months | 20 | 8.1 | 9 | 9.0 | 1.74 (0.69, 4.42) | 0.24 |  | 15 | 19.2 | 2 | 9.1 | 0.92 (0.14, 6.16) | 0.93 |  | 2 | 3.2 | 4 | 8.9 | 0.57 (0.06, 5.54) | 0.63 |  | 4 | 4.8 | 2 | 6.3 | 6.93 (0.69, 69.10) | 0.10 |  | 10 | 10.6 | 5 | 10.2 | 1.82 (0.45, 7.44) | 0.40 |
| ≥4 children & ≥62 months | 61 | 24.8 | 34 | 34.0 | 1.62 (0.87, 3.02) | 0.13 |  | 22 | 28.2 | 12 | 54.5 | 3.00 (0.86, 10.50) | 0.09 |  | 31 | 49.2 | 18 | 40.0 | 0.69 (0.20, 2.36) | 0.55 |  | 37 | 44.6 | 20 | 62.5 | 2.74 (0.79, 9.50) | 0.11 |  | 41 | 43.6 | 30 | 61.2 | 2.79 (0.98, 7.89) | 0.054 |
| Trend‡ |  |  |  |  | 1.19 (0.97, 1.46) | 0.092 |  |  |  |  |  | 1.42 (0.93, 2.16) | 0.10 |  |  |  |  |  | 0.88 (0.59, 1.33) | 0.55 |  |  |  |  |  | 1.39 (0.93, 2.09) | 0.11 |  |  |  |  |  | 1.39 (0.996, 1.95) | 0.053 |

† Point estimates and 95% confidence intervals were from multivariable models, adjusting for categorized age at diagnosis and BMI, except where noticed. Estimates of numbers of children, cumulative and averaged breastfeeding duration, and combined age at first pregnancy and number of children were computed among parous women. ‡ Results were from the trend analysis using the categorical risk factor as a trend. ᵃ Nulliparous cases were women who reported never pregnant, never given birth, and had no children (N=37, 4.4%). AKU, Aga Khan University; BMI, body mass index; CI, confidence interval; ER, estrogen receptor; *N*, results not shown due to limited sample sizes; OR, odds ratio; T, tertile.
