# Supplementary material for: Breast cancer risk factors in relation to molecular subtypes in breast cancer patients from Kenya
Source: Breast Cancer Res. 2021 Jun 26;23:68. doi: 10.1186/s13058-021-01446-3 (PMC8235821; doi:10.1186/s13058-021-01446-3)
Supplement: Supplementary file 4 — Supplementary Table 4. Associations between BMI and HER2 status stratified by hospitals (N=821) [file 13058_2021_1446_MOESM4_ESM.docx]

**Supplementary Table 4. Associations between BMI and HER2 status stratified by hospitals (N=821*)**

|  | **Hospitals** | | | | | | | | | | | | | | | | | | | | | | | | | | | | | | | | | |
| --- | --- | --- | --- | --- | --- | --- | --- | --- | --- | --- | --- | --- | --- | --- | --- | --- | --- | --- | --- | --- | --- | --- | --- | --- | --- | --- | --- | --- | --- | --- | --- | --- | --- | --- |
|  | **AKU (n=345, 42%)** | | | | | |  | **Kijabe (n=104, 13%)** | | | | | |  | **Nyeri (n=106, 13%)** | | | | | |  | **St Mary's (n=122, 15%)** | | | | | |  | **Others (n=144, 18%)** | | | | | |
|  | **HER2-** | | **HER2+** | | **HER2- vs. HER2+** | |  | **HER2-** | | **HER2+** | | **HER2- vs. HER2+** | |  | **HER2-** | | **HER2+** | | **HER2- vs. HER2+** | |  | **HER2-** | | **HER2+** | | **HER2- vs. HER2+** | |  | **HER2-** | | **HER2+** | | **HER2- vs. HER2+** | |
|  | **N** | **%** | **N** | **%** | **OR (95% CI)†** | **P†** |  | **N** | % | **N** | % | **OR (95% CI)†** | **P†** |  | **N** | % | **N** | % | **OR (95% CI)†** | **P†** |  | **N** | % | **N** | % | **OR (95% CI)†** | **P†** |  | **N** | % | **N** | % | **OR (95% CI)†** | **P†** |
| **BMI** |  |  |  |  |  |  |  |  |  |  |  |  |  |  |  |  |  |  |  |  |  |  |  |  |  |  |  |  |  |  |  |  |  |  |
| Normal (<25.0) | 36 | 16.3 | 29 | 32.6 | Ref |  |  | 25 | 41.0 | 9 | 36.0 | Ref |  |  | 17 | 38.6 | 6 | 37.5 | Ref |  |  | 30 | 41.7 | 6 | 35.3 | Ref |  |  | 31 | 39.2 | 14 | 45.2 | Ref |  |
| Overweight (25.0 - 29.9) | 87 | 39.4 | 36 | 40.4 | **0.52 (0.28, 0.97)** | **0.041** |  | 24 | 39.3 | 9 | 36.0 | 1.07 (0.36, 3.16) | 0.91 |  | 19 | 43.2 | 8 | 50.0 | 1.31 (0.37, 4.65) | 0.68 |  | 27 | 37.5 | 10 | 58.8 | 2.10 (0.66, 6.71) | 0.21 |  | 30 | 38.0 | 7 | 22.6 | 0.53 (0.18, 1.58) | 0.26 |
| Obese (≥30.0) | 98 | 44.3 | 24 | 27.0 | **0.31 (0.16, 0.60)** | **0.0006** |  | 12 | 19.7 | 7 | 28.0 | 1.56 (0.46, 5.24) | 0.47 |  | 8 | 18.2 | 2 | 12.5 | 0.80 (0.13, 5.00) | 0.81 |  | 15 | 20.8 | 1 | 5.9 | 0.38 (0.04, 3.55) | 0.40 |  | 18 | 22.8 | 10 | 32.3 | 1.26 (0.45, 3.52) | 0.66 |
| Trend‡ |  |  |  |  | **0.56 (0.40, 0.78)** | **0.0006** |  |  |  |  |  | 1.23 (0.67, 2.26) | 0.50 |  |  |  |  |  | 0.98 (0.43, 2.23) | 0.95 |  |  |  |  |  | 0.92 (0.44, 1.95) | 0.83 |  |  |  |  |  | 1.10 (0.64, 1.87) | 0.74 |

* Seventeen patients were missing for their HER2 status. † Point estimates and 95% confidence intervals were from multivariable models, adjusting for categorized age at diagnosis. ‡ Results were from the trend analysis using the categorical BMI as a trend. AKU, Aga Khan University; BMI, body mass index; CI, confidence interval; HER2, human epidermal growth factor receptor-2; OR, odds ratio.
