# Supplementary material for: Breast cancer risk factors in relation to molecular subtypes in breast cancer patients from Kenya
Source: Breast Cancer Res. 2021 Jun 26;23:68. doi: 10.1186/s13058-021-01446-3 (PMC8235821; doi:10.1186/s13058-021-01446-3)
Supplement: Supplementary file 5 — Supplementary Table 5. Associations between key risk factors and ER status by age at diagnosis (N=834) [file 13058_2021_1446_MOESM5_ESM.docx]

| **Supplementary Table 5. Associations between key risk factors and ER status by age at diagnosis (N=834*)** | | | | | | | | | | | | |  | |
| --- | --- | --- | --- | --- | --- | --- | --- | --- | --- | --- | --- | --- | --- | --- |
|  | **Age at diagnosis <50 year (n= 451, 54.1%)** | | | | | |  | **Age at diagnosis ≥50 year (n= 383, 45.9%)** | | | | | |  |
|  | **ER+** | | **ER-** | | **ER- vs. ER+** | |  | **ER+** | | **ER-** | | **ER- vs. ER+** | |  |
|  | **N** | **%** | **N** | **%** | **OR (95% CI)†** | ***P*†** |  | **N** | % | **N** | % | **OR (95% CI)†** | ***P*†** |  |
| **BMI** |  |  |  |  |  |  |  |  |  |  |  |  |  |  |
| Normal (<25.0) | 93 | 35.4 | 48 | 42.9 | 1.00 (Ref) |  |  | 43 | 35.4 | 26 | 42.9 | 1.00 (Ref) |  |  |
| Overweight (25.0 - 29.9) | 109 | 41.4 | 33 | 29.5 | 0.58 (0.31, 1.07) | 0.08 |  | 74 | 41.4 | 46 | 29.5 | 1.26 (0.58, 2.74) | 0.56 |  |
| Obese (≥30.0) | 61 | 23.2 | 31 | 27.7 | 1.22 (0.63, 2.39) | 0.55 |  | 75 | 23.2 | 30 | 27.7 | 0.65 (0.28, 1.47) | 0.30 |  |
| Trend‡ |  |  |  |  | 1.05 (0.75, 1.47) | 0.78 |  |  |  |  |  | 0.77 (0.52, 1.14) | 0.19 |  |
| **Age at menarche/year** |  |  |  |  |  |  |  |  |  |  |  |  |  |  |
| ≤13 (9-13) | 86 | 27.4 | 35 | 27.1 | 1.00 (Ref) |  |  | 55 | 27.4 | 22 | 27.1 | 1.00 (Ref) |  |  |
| 14 | 74 | 23.6 | 41 | 31.8 | 1.53 (0.77, 3.04) | 0.23 |  | 64 | 23.6 | 30 | 31.8 | 2.04 (0.86, 4.84) | 0.11 |  |
| ≥15 (15-20) | 154 | 49.0 | 53 | 41.1 | 0.98 (0.52, 1.87) | 0.96 |  | 121 | 49.0 | 65 | 41.1 | **2.25 (1.04, 4.84)** | **0.038** |  |
| Trend‡ |  |  |  |  | 0.97 (0.71, 1.32) | 0.82 |  |  |  |  |  | 1.44 (0.997, 2.07) | 0.052 |  |
| **Age at first pregnancy/year** |  |  |  |  |  |  |  |  |  |  |  |  |  |  |
| <20 | 63 | 19.7 | 44 | 34.1 | 1.00 (Ref) |  |  | 66 | 19.7 | 42 | 34.1 | 1.00 (Ref) |  |  |
| 20-24 | 140 | 43.8 | 57 | 44.2 | 0.77 (0.38, 1.57) | 0.47 |  | 113 | 43.8 | 51 | 44.2 | 0.57 (0.27, 1.20) | 0.14 |  |
| 25-29 | 72 | 22.5 | 16 | 12.4 | 0.48 (0.18, 1.26) | 0.14 |  | 43 | 22.5 | 19 | 12.4 | 0.70 (0.26, 1.89) | 0.48 |  |
| Nulliparousᵃ or ≥30 | 45 | 14.1 | 12 | 9.3 | 0.98 (0.30, 3.15) | 0.97 |  | 25 | 14.1 | 11 | 9.3 | 1.15 (0.26, 5.16) | 0.85 |  |
| Trend‡ |  |  |  |  | 0.88 (0.61, 1.27) | 0.49 |  |  |  |  |  | 0.94 (0.62, 1.43) | 0.78 |  |
| **Parity** |  |  |  |  |  |  |  |  |  |  |  |  |  |  |
| Nulliparousᵃ | 24 | 7.5 | 5 | 3.8 | 0.38 (0.07, 2.11) | 0.27 |  | 12 | 7.5 | 124 | 3.8 | 0.38 (0.04, 3.42) | 0.39 |  |
| Parous | 297 | 92.5 | 125 | 96.2 | 1.00 (Ref) |  |  | 245 | 92.5 | 19 | 96.2 | 1.00 (Ref) |  |  |
| **Number of children** |  |  |  |  |  |  |  |  |  |  |  |  |  |  |
| 1 or 2 | 127 | 42.8 | 38 | 30.4 | 1.00 (Ref) |  |  | 43 | 17.6 | 17 | 13.7 | 1.00 (Ref) |  |  |
| 3 or 4 | 132 | 44.4 | 58 | 46.4 | 1.54 (0.82, 2.90) | 0.18 |  | 85 | 34.7 | 32 | 25.8 | 0.98 (0.37, 2.59) | 0.98 |  |
| ≥ 5 | 38 | 12.8 | 29 | 23.2 | **2.87 (1.20, 6.87)** | **0.018** |  | 117 | 47.8 | 75 | 60.5 | 1.74 (0.67, 4.54) | 0.26 |  |
| Trend‡ |  |  |  |  | **1.67 (1.09, 2.57)** | **0.019** |  |  |  |  |  | 1.40 (0.88, 2.23) | 0.16 |  |
| **Cumulative breastfeeding**  **duration /monthᵇ** |  |  |  |  |  |  |  |  |  |  |  |  |  |  |
| Q1: 1 - <39 | 104 | 35.6 | 29 | 23.4 | 1.00 (Ref) |  |  | 48 | 20.7 | 10 | 8.6 | 1.00 (Ref) |  |  |
| Q2: 39 - <62 | 88 | 30.1 | 40 | 32.3 | 1.82 (0.89, 3.72) | 0.10 |  | 38 | 16.4 | 25 | 21.4 | **5.41 (1.60, 18.34)** | **0.007** |  |
| Q3: 62 - <96 | 63 | 21.6 | 31 | 25.0 | 1.05 (0.45, 2.45) | 0.91 |  | 61 | 26.3 | 27 | 23.1 | 2.65 (0.73, 9.58) | 0.14 |  |
| Q4: ≥96 | 37 | 12.7 | 24 | 19.4 | 0.99 (0.34, 2.89) | 0.98 |  | 85 | 36.6 | 55 | 47.0 | 3.17 (0.86, 11.73) | 0.08 |  |
| Trend‡ |  |  |  |  | 0.998 (0.72, 1.39) | 0.99 |  |  |  |  |  | 1.16 (0.81, 1.67) | 0.41 |  |
| **Mean breastfeeding duration**  **per child/month** |  |  |  |  |  |  |  |  |  |  |  |  |  |  |
|  |  |  |  |  |  |  |  |  |  |  |  |  |  |  |
| <12 | 41 | 14.0 | 16 | 12.9 | 1.00 (Ref) |  |  | 43 | 18.5 | 20 | 17.1 | 1.00 (Ref) |  |  |
| 12- 23 | 162 | 55.5 | 68 | 54.8 | 0.78 (0.36, 1.72) | 0.54 |  | 113 | 48.7 | 62 | 53.0 | 1.69 (0.70, 4.10) | 0.24 |  |
| ≥ 24 | 89 | 30.5 | 40 | 32.3 | 1.22 (0.51, 2.89) | 0.66 |  | 76 | 32.8 | 35 | 29.9 | 1.34 (0.52, 3.45) | 0.55 |  |
| Trend‡ |  |  |  |  | 1.18 (0.77, 1.81) | 0.45 |  |  |  |  |  | 1.07 (0.69, 1.67) | 0.76 |  |
| **Age at first pregnancy &**  **Number of children** |  |  |  |  |  |  |  |  |  |  |  |  |  |  |
| Age 25+ yr, 1-3 births | 79 | 26.7 | 19 | 16.2 | 1.00 (Ref) |  |  | 19 | 15.2 | 18 | 14.9 | 1.00 (Ref) |  |  |
| Age <25 yr, 1-3 births | 123 | 41.6 | 54 | 21.3 | 1.57 (0.74, 3.33) | 0.24 |  | 54 | 43.2 | 17 | 14.1 | 0.65 (0.23, 1.80) | 0.40 |  |
| Age 25+ yr, 4+ births | 15 | 5.1 | 5 | 8.1 | 1.80 (0.46, 7.04) | 0.40 |  | 5 | 4.0 | 10 | 8.3 | 1.12 (0.32, 4.00) | 0.86 |  |
| Age <25 yr, 4+ births | 79 | 26.7 | 47 | 54.5 | 1.91 (0.81, 4.50) | 0.14 |  | 47 | 37.6 | 76 | 62.8 | 1.36 (0.56, 3.33) | 0.50 |  |
| Trend‡ |  |  |  |  | 1.19 (0.92, 1.54) | 0.19 |  |  |  |  |  | 1.19 (0.90, 1.57) | 0.22 |  |
| **Number of children & Cumulative breastfeeding duration** |  |  |  |  |  |  |  |  |  |  |  |  |  |  |
| Nulliparous or ≤3 children &  <62 months | 191 | 60.4 | 65 | 50.4 | 1.00 (Ref) |  |  | 72 | 29.5 | 24 | 20.2 | 1.00 (Ref) |  |  |
| ≤3 children & ≥62 months | 31 | 9.8 | 12 | 9.3 | 0.98 (0.40, 2.44) | 0.97 |  | 25 | 10.2 | 11 | 9.2 | 0.99 (0.34, 2.86) | 0.98 |  |
| ≥4 children & <62 months | 25 | 7.9 | 9 | 7.0 | 1.10 (0.40, 3.02) | 0.85 |  | 26 | 10.7 | 13 | 10.9 | 2.44 (0.75, 7.93) | 0.14 |  |
| ≥4 children & ≥62 months | 69 | 21.8 | 43 | 33.3 | 1.32 (0.67, 2.59) | 0.42 |  | 121 | 49.6 | 71 | 59.7 | 1.80 (0.81, 3.99) | 0.15 |  |
| Trend‡ |  |  |  |  | 1.09 (0.88, 1.36) | 0.44 |  |  |  |  |  | 1.22 (0.94, 1.59) | 0.13 |  |

* Four cancer patients had missing data for age at diagnosis and therefore were not included in stratification analyses. † Point estimates and confidence interval were from multivariable logistic regression models, adjusting for BMI, age at menarche, age at first pregnancy, number of children, mean breastfeeding duration per child, age at menopause, family history of breast cancer in first degree female relative, occupation, education level, and hospitals, except where noticed. ‡ Results were from the trend analysis using the categorical risk factor as a trend. ᵃ Women who reported never pregnant, never gave birth, and had no child were categorized as "Nulliparous" in modeling analyses. BMI, body mass index; CI, confidence interval; ER, estrogen receptor; OR, odds ratio; Q, quartile.
