# Supplementary material for: Breast cancer risk factors in relation to molecular subtypes in breast cancer patients from Kenya
Source: Breast Cancer Res. 2021 Jun 26;23:68. doi: 10.1186/s13058-021-01446-3 (PMC8235821; doi:10.1186/s13058-021-01446-3)
Supplement: Supplementary file 6 — Supplementary Table 6. Associations between breast cancer risk factors and tumor molecular subtypes in Kenyan breast cancer patients (N=776) [file 13058_2021_1446_MOESM6_ESM.docx]

**Supplementary Table 6. Associations between breast cancer risk factors and tumor molecular subtypes in Kenyan breast cancer patients (N=776*)**

|  | **Tumor subtypes** | | | | | | | | | | | | | | | | | | | | | | | | | | |
| --- | --- | --- | --- | --- | --- | --- | --- | --- | --- | --- | --- | --- | --- | --- | --- | --- | --- | --- | --- | --- | --- | --- | --- | --- | --- | --- | --- |
|  | **Luminal A n=286** | |  | **Luminal B HER2- n=157** | | **Luminal B HER2-  vs. Luminal A** | |  | **Luminal B HER2+ n=137** | | **Luminal B HER2+  vs. Luminal A** | |  | **HER2-enriched  n=88** | | **HER2-enriched  vs. Luminal A** | |  | **Core-basal like  n=57** | | **Core-basal like  vs. Luminal A** | |  | **Five-negative n=51** | | **Five-negative  vs. Luminal A** | |
|  | **N** | **%** |  | **N** | **%** | **OR (95% CI)†** | ***P†*** |  | **N** | **%** | **OR (95% CI)†** | ***P†*** |  | **N** | **%** | **OR (95% CI)†** | ***P†*** |  | **N** | **%** | **OR (95% CI)†** | ***P†*** |  | **N** | **%** | **OR (95% CI)†** | ***P†*** |
| **Age at diagnosis/year** |  |  |  |  |  |  |  |  |  |  |  |  |  |  |  |  |  |  |  |  |  |  |  |  |  |  |  |
| < 50 | 142 | 50.2 |  | 96 | 61.5 | 1.00 (Ref) |  |  | 84 | 61.3 | 1.00 (Ref) |  |  | 44 | 50.0 | 1.00 (Ref) |  |  | 32 | 56.1 | 1.00 (Ref) |  |  | 27 | 52.9 | 1.00 (Ref) |  |
| ≥ 50 | 141 | 49.8 |  | 60 | 38.5 | 0.54 (0.19, 1.59) | 0.27 |  | 53 | 38.7 | 1.52 (0.49, 4.71) | 0.46 |  | 44 | 50.0 | 2.04 (0.61, 6.88) | 0.25 |  | 25 | 43.9 | 0.27 (0.06, 1.25) | 0.09 |  | 24 | 47.1 | **0.24 (0.06, 0.95)** | **0.043** |
| **BMI/ kg/m^2^** |  |  |  |  |  |  |  |  |  |  |  |  |  |  |  |  |  |  |  |  |  |  |  |  |  |  |  |
| Normal (<25.0) | 60 | 27.3 |  | 36 | 28.8 | 1.00 (Ref) |  |  | 35 | 32.4 | 1.00 (Ref) |  |  | 29 | 41.4 | 1.00 (Ref) |  |  | 18 | 37.5 | 1.00 (Ref) |  |  | 14 | 32.6 | 1.00 (Ref) |  |
| Overweight (25.0 - 29.9) | 84 | 38.2 |  | 54 | 43.2 | 1.15 (0.63, 2.11) | 0.65 |  | 45 | 41.7 | 0.99 (0.53, 1.84) | 0.97 |  | 25 | 35.7 | 0.57 (0.29, 1.16) | 0.12 |  | 12 | 25.0 | 0.59 (0.23, 1.53) | 0.28 |  | 18 | 41.9 | 0.63 (0.26, 1.55) | 0.32 |
| Obese (≥30.0) | 76 | 34.5 |  | 35 | 28.0 | 0.79 (0.40, 1.55) | 0.49 |  | 28 | 25.9 | 0.71 (0.35, 1.42) | 0.33 |  | 16 | 22.9 | 0.37 (0.16, 0.83) | **0.016** |  | 18 | 37.5 | 0.87 (0.35, 2.18) | 0.77 |  | 11 | 25.6 | 0.47 (0.17, 1.29) | 0.14 |
| Trend‡ |  |  |  |  |  | 0.88 (0.63, 1.24) | 0.47 |  |  |  | 0.84 (0.60, 1.19) | 0.33 |  |  |  | **0.60 (0.40, 0.90)** | **0.014** |  |  |  | 0.95 (0.59, 1.53) | 0.84 |  |  |  | 0.68 (0.41, 1.14) | 0.14 |
| **Age at menarche/year** |  |  |  |  |  |  |  |  |  |  |  |  |  |  |  |  |  |  |  |  |  |  |  |  |  |  |  |
| ≤13 (9-13) | 69 | 25.7 |  | 38 | 24.8 | 1.00 (Ref) |  |  | 33 | 24.8 | 1.00 (Ref) |  |  | 23 | 26.4 | 1.00 (Ref) |  |  | 7 | 13.5 | 1.00 (Ref) |  |  | 14 | 28.0 | 1.00 (Ref) |  |
| 14 | 68 | 25.4 |  | 34 | 22.2 | 1.14 (0.57, 2.29) | 0.71 |  | 35 | 26.3 | 1.40 (0.70, 2.79) | 0.34 |  | 26 | 29.9 | 1.65 (0.72, 3.74) | 0.23 |  | 15 | 28.8 | 2.01 (0.69, 5.89) | 0.20 |  | 12 | 24.0 | 1.37 (0.50, 3.76) | 0.54 |
| ≥15 (15-20) | 131 | 48.9 |  | 81 | 52.9 | 1.45 (0.79, 2.64) | 0.23 |  | 65 | 48.9 | 1.09 (0.58, 2.03) | 0.79 |  | 38 | 43.7 | 1.31 (0.63, 2.71) | 0.47 |  | 30 | 57.7 | 2.11 (0.80, 5.56) | 0.13 |  | 24 | 48.0 | 1.30 (0.52, 3.24) | 0.58 |
| Trend‡ |  |  |  |  |  | 1.22 (0.90, 1.65) | 0.20 |  |  |  | 1.02 (0.75, 1.39) | 0.89 |  |  |  | 1.11 (0.78, 1.58) | 0.56 |  |  |  | 1.39 (0.88, 2.19) | 0.15 |  |  |  | 1.12 (0.72, 1.76) | 0.62 |
| **Age at first pregnancy/year** |  |  |  |  |  |  |  |  |  |  |  |  |  |  |  |  |  |  |  |  |  |  |  |  |  |  |  |
| <20 | 72 | 26.2 |  | 28 | 18.1 | 1.00 (Ref) |  |  | 30 | 21.9 | 1.00 (Ref) |  |  | 31 | 35.6 | 1.00 (Ref) |  |  | 18 | 32.1 | 1.00 (Ref) |  |  | 19 | 37.3 | 1.00 (Ref) |  |
| 20-24 | 121 | 44.0 |  | 80 | 51.6 | **2.24 (1.09, 4.61)** | **0.029** |  | 53 | 38.7 | 0.95 (0.46, 1.99) | 0.90 |  | 30 | 34.5 | 0.63 (0.29, 1.36) | 0.24 |  | 25 | 44.6 | 0.85 (0.34, 2.17) | 0.74 |  | 26 | 51.0 | 0.54 (0.22, 1.37) | 0.19 |
| 25-29 | 51 | 18.5 |  | 31 | 20.0 | 2.42 (0.98, 5.97) | 0.055 |  | 31 | 22.6 | 1.29 (0.53, 3.16) | 0.57 |  | 16 | 18.4 | 0.91 (0.33, 2.50) | 0.86 |  | 9 | 16.1 | 1.07 (0.30, 3.77) | 0.92 |  | 4 | 7.8 | **0.24 (0.06, 0.99)** | **0.049** |
| Nulliparous^a^ or age ≥30 | 31 | 11.3 |  | 16 | 10.3 | 1.05 (0.29, 3.74) | 0.94 |  | 23 | 16.8 | 1.00 (0.31, 3.24) | 0.99 |  | 10 | 11.5 | 1.23 (0.31, 4.82) | 0.77 |  | 4 | 7.1 | 0.90 ((0.17, 4.64) | 0.90 |  | 2 | 3.9 | **0.03 (0.001, 0.76)** | **0.034** |
| Trend‡ |  |  |  |  |  | 1.13 (0.80, 1.58) | 0.50 |  |  |  | 1.08 (0.76, 1.55) | 0.66 |  |  |  | 1.02 (0.66 1.57) | 0.92 |  |  |  | 0.996 (0.60, 1.65) | 0.99 |  |  |  | **0.43 (0.23, 0.78)** | **0.006** |
| **Parity** |  |  |  |  |  |  |  |  |  |  |  |  |  |  |  |  |  |  |  |  |  |  |  |  |  |  |  |
| Nulliparous^a^ | 14 | 4.9 |  | 8 | 5.1 | 1.01 (0.20, 5.21) | 0.99 |  | 12 | 8.8 | 0.99 (0.23, 4.30) | 0.99 |  | 4 | 4.5 | 0.53 (0.06, 4.64) | 0.57 |  | 0 | 0.0 | *--* | *--* |  | 1 | 2.0 | 18.99 (0.46, 785.28) | 0.12 |
| Parous |  |  |  |  |  | 1.00 (Ref) |  |  |  |  | 1.00 (Ref) |  |  |  |  | 1.00 (Ref) |  |  |  |  | 1.00 (Ref) |  |  |  |  | 1.00 (Ref) |  |
| **Number of children** |  |  |  |  |  |  |  |  |  |  |  |  |  |  |  |  |  |  |  |  |  |  |  |  |  |  |  |
| 1 or 2 | 73 | 26.8 |  | 52 | 34.9 | 1.00 (Ref) |  |  | 49 | 39.2 | 1.00 (Ref) |  |  | 19 | 22.6 | 1.00 (Ref) |  |  | 13 | 22.8 | 1.00 (Ref) |  |  | 11 | 22.0 | 1.00 (Ref) |  |
| 3 or 4 | 113 | 41.5 |  | 55 | 36.9 | **0.49 (0.26, 0.91)** | **0.023** |  | 46 | 36.8 | **0.42 (0.22, 0.81)** | **0.010** |  | 26 | 31.0 | 0.83 (0.36, 1.93) | 0.67 |  | 22 | 38.6 | 1.17 (0.44, 3.11) | 0.75 |  | 21 | 42.0 | 2.11 (0.70, 6.37) | 0.19 |
| ≥ 5 | 86 | 31.6 |  | 42 | 28.2 | 0.48 (0.22, 1.04) | 0.062 |  | 30 | 24.0 | **0.39 (0.17, 0.91)** | **0.030** |  | 39 | 46.4 | 1.66 (0.63, 4.37) | 0.30 |  | 22 | 38.6 | 1.47 (0.45, 4.79) | 0.53 |  | 18 | 36.0 | 2.15 (0.55, 8.32) | 0.27 |
| Trend‡ |  |  |  |  |  | **0.67 (0.46, 0.99)** | **0.044** |  |  |  | **0.60 (0.40, 0.92)** | **0.017** |  |  |  | 1.33 (0.81, 2.17) | 0.26 |  |  |  | 1.21 (0.67, 2.16) | 0.53 |  |  |  | 1.40 (0.74, 2.63) | 0.30 |
| **Cumulative breastfeeding**  **duration/month^b^** |  |  |  |  |  |  |  |  |  |  |  |  |  |  |  |  |  |  |  |  |  |  |  |  |  |  |  |
| Q1: 1 - <39 | 64 | 24.3 |  | 49 | 34.0 | 1.00 (Ref) |  |  | 39 | 32.2 | 1.00 (Ref) |  |  | 15 | 18.3 | 1.00 (Ref) |  |  | 9 | 16.4 | 1.00 (Ref) |  |  | 5 | 10.4 | 1.00 (Ref) |  |
| Q2: 39 - <62 | 59 | 22.4 |  | 38 | 26.4 | 0.93 (0.46, 1.88) | 0.84 |  | 30 | 24.8 | 1.08 (0.52, 2.26) | 0.84 |  | 22 | 26.8 | **2.59 (1.00, 6.69)** | **0.049** |  | 17 | 30.9 | **4.61 (1.32, 16.14)** | **0.017** |  | 14 | 29.2 | 3.13 (0.76, 12.82) | 0.11 |
| Q3: 62 - <96 | 71 | 27.0 |  | 24 | 16.7 | 0.48 (0.20, 1.14) | 0.10 |  | 29 | 24.0 | 0.88 (0.37, 2.08) | 0.76 |  | 14 | 17.1 | 0.79 (0.26, 2.38) | 0.67 |  | 12 | 21.8 | 1.43 (0.33, 6.11) | 0.63 |  | 15 | 31.3 | 3.05 (0.68, 13.65) | 0.15 |
| Q4: ≥96 | 69 | 26.2 |  | 33 | 22.9 | 0.87 (0.33, 2.27) | 0.78 |  | 23 | 19.0 | 1.01 (0.35, 2.90) | 0.98 |  | 31 | 37.8 | 1.27 (0.39, 4.09) | 0.69 |  | 17 | 30.9 | 2.81 (0.59, 13.38) | 0.19 |  | 14 | 29.2 | 3.65 (0.70, 19.12) | 0.13 |
| Trend‡ |  |  |  |  |  | 0.88 (0.64, 1.21) | 0.43 |  |  |  | 0.97 (0.69, 1.35) | 0.85 |  |  |  | 0.96 (0.66, 1.39) | 0.83 |  |  |  | 1.16 (0.73, 1.85) | 0.53 |  |  |  | 1.41 (0.87, 2.29) | 0.16 |
| **Mean breastfeeding**  **duration per child/month** |  |  |  |  |  |  |  |  |  |  |  |  |  |  |  |  |  |  |  |  |  |  |  |  |  |  |  |
| <12 | 34 | 12.9 |  | 27 | 18.8 | 1.00 (Ref) |  |  | 21 | 17.4 | 1.00 (Ref) |  |  | 11 | 13.4 | 1.00 (Ref) |  |  | 10 | 18.2 | 1.00 (Ref) |  |  | 5 | 10.4 | 1.00 (Ref) |  |
| 12- 23 | 142 | 54.0 |  | 72 | 50.0 | 0.61 (0.29, 1.30) | 0.20 |  | 67 | 55.4 | 0.67 (0.31, 1.43) | 0.30 |  | 49 | 59.8 | 1.29 (0.48, 3.41) | 0.61 |  | 26 | 47.3 | 0.72 (0.24, 2.13) | 0.55 |  | 23 | 47.9 | 1.19 (0.31, 4.62) | 0.80 |
| ≥ 24 | 87 | 33.1 |  | 45 | 31.3 | 0.77 (0.34, 1.71) | 0.52 |  | 33 | 27.3 | 0.48 (0.20, 1.14) | 0.10 |  | 22 | 26.8 | 1.21 (0.42, 3.54) | 0.72 |  | 19 | 34.5 | 0.94 (0.29, 2.99) | 0.91 |  | 20 | 41.7 | 3.37 (0.84, 13.58) | 0.09 |
| Trend‡ |  |  |  |  |  | 0.95 (0.64, 1.40) | 0.78 |  |  |  | 0.70 (0.47, 1.07) | 0.10 |  |  |  | 1.06 (0.65, 1.72) | 0.82 |  |  |  | 1.03 (0.58, 1.82) | 0.92 |  |  |  | **2.16 (1.13, 4.14)** | **0.020** |
| **Age at first pregnancy &**  **Number of children** |  |  |  |  |  |  |  |  |  |  |  |  |  |  |  |  |  |  |  |  |  |  |  |  |  |  |  |
| Age 25+ yr, 1-3 births | 54 | 20.7 |  | 33 | 22.4 | 1.00 (Ref) |  |  | 31 | 24.8 | 1.00 (Ref) |  |  | 15 | 17.9 | 1.00 (Ref) |  |  | 10 | 17.9 | 1.00 (Ref) |  |  | 5 | 10.0 | 1.00 (Ref) |  |
| Age <25 yr, 1-3 births | 80 | 30.7 |  | 53 | 36.1 | 1.01 (0.50, 2.03) | 0.98 |  | 40 | 32.0 | 0.84 (0.41, 1.72) | 0.62 |  | 19 | 22.6 | 0.82 (0.31, 2.14) | 0.68 |  | 17 | 30.4 | 0.87 (0.29, 2.60) | 0.81 |  | 21 | 42.0 | **3.68 (1.04, 13.04)** | **0.044** |
| Age 25+ yr, 4+ births | 15 | 5.7 |  | 7 | 4.8 | 0.52 (0.14, 1.91) | 0.32 |  | 11 | 8.8 | 1.05 (0.34, 3.26) | 0.94 |  | 8 | 9.5 | 2.04 (0.57, 7.33) | 0.28 |  | 3 | 5.4 | 1.27 (0.20, 8.00) | 0.80 |  | 0 | 0.0 | -- | -- |
| Age <25 yr, 4+ births | 112 | 42.9 |  | 54 | 36.7 | 0.54 (0.25, 1.18) | 0.12 |  | 43 | 34.4 | 0.55 (0.24, 1.23) | 0.14 |  | 42 | 50.0 | 1.26 (0.49, 3.30) | 0.63 |  | 26 | 46.4 | 1.08 (0.34, 3.42) | 0.90 |  | 24 | 48.0 | 3.84 (0.96, 15.34) | 0.057 |
| Trend‡ |  |  |  |  |  | 0.79 (0.62, 1.01) | 0.055 |  |  |  | 0.83 (0.64, 1.07) | 0.15 |  |  |  | 1.14 (0.85, 1.54) | 0.38 |  |  |  | 1.06 (0.74, 1.52) | 0.76 |  |  |  | 1.29 (0.88, 1.88) | 0.19 |
| **Number of children & Cumulative breastfeeding duration** |  |  |  |  |  |  |  |  |  |  |  |  |  |  |  |  |  |  |  |  |  |  |  |  |  |  |  |
| Nulliparous^a^ or ≤3 children &  <62 months | 117 | 42.24 |  | 78 | 51.3 | 1.00 (Ref) |  |  | 68 | 51.1 | 1.00 (Ref) |  |  | 31 | 36.0 | 1.00 (Ref) |  |  | 21 | 38.2 | 1.00 (Ref) |  |  | 18 | 36.7 | 1.00 (Ref) |  |
| ≤3 children & ≥62 months | 30 | 10.83 |  | 13 | 8.6 | 0.58 (0.24, 1.38) | 0.22 |  | 12 | 9.0 | 0.61 (0.26, 1.43) | 0.26 |  | 6 | 7.0 | 0.38 (0.10, 1.45) | 0.16 |  | 5 | 9.1 | 0.66 (0.17, 2.57) | 0.55 |  | 8 | 16.3 | 2.39 (0.83, 6.83) | 0.10 |
| ≥4 children & <62 months | 20 | 7.22 |  | 17 | 11.2 | 0.67 (0.24, 1.87) | 0.45 |  | 13 | 9.8 | 1.02 (0.38, 2.74) | 0.96 |  | 10 | 11.6 | 2.06 (0.72, 5.92) | 0.18 |  | 5 | 9.1 | 1.57 (0.40, 6.15) | 0.52 |  | 2 | 4.1 | 1.19 (0.22, 6.31) | 0.84 |
| ≥4 children & ≥62 months | 110 | 39.71 |  | 44 | 28.9 | **0.44 (0.24, 0.82)** | **0.01** |  | 40 | 30.1 | 0.52 (0.27, 1.02) | 0.056 |  | 39 | 45.3 | 1.16 (0.56, 2.43) | 0.69 |  | 24 | 43.6 | 1.14 (0.46, 2.85) | 0.78 |  | 21 | 42.9 | 1.51 (0.58, 3.95) | 0.40 |
| Trend‡ |  |  |  |  |  | **0.76 (0.62, 0.94)** | **0.011** |  |  |  | 0.82 (0.66, 1.02) | 0.07 |  |  |  | 1.09 (0.85, 1.39) | 0.52 |  |  |  | 1.06 (0.79, 1.44) | 0.69 |  |  |  | 1.12 (0.83, 1.52) | 0.47 |
| **Menopausal status^c^** |  |  |  |  |  |  |  |  |  |  |  |  |  |  |  |  |  |  |  |  |  |  |  |  |  |  |  |
| Premenopausal | 144 | 50.3 |  | 91 | 58.0 | 1.00 (Ref) |  |  | 83 | 61.0 | 1.00 (Ref) |  |  | 44 | 50.0 | 1.00 (Ref) |  |  | 28 | 49.1 | 1.00 (Ref) |  |  | 22 | 44.0 | 1.00 (Ref) |  |
| Postmenopausal | 142 | 49.7 |  | 66 | 42.0 | 1.06 (0.42, 2.67) | 0.89 |  | 53 | 39.0 | 0.40 (0.14, 1.16) | 0.09 |  | 44 | 50.0 | 0.64 (0.20, 2.06) | 0.45 |  | 29 | 50.9 | 2.27 (0.69, 7.50) | 0.18 |  | 28 | 56.0 | 3.07 (0.94, 10.05) | 0.064 |
| **Age at menopause/year^d^** |  |  |  |  |  |  |  |  |  |  |  |  |  |  |  |  |  |  |  |  |  |  |  |  |  |  |  |
| Premenopausal | 144 | 50.3 |  | 91 | 58.0 | *--* | |  | 83 | 61.0 | -- | |  | 44 | 50.0 | *--* | |  | 28 | 49.1 | *--* | |  | 22 | 44.0 | *--* | |
| < 50 | 58 | 22.5 |  | 27 | 18.5 | 1.00 (Ref) |  |  | 31 | 23.5 | 1.00 (Ref) |  |  | 85 | 33.7 | 1.00 (Ref) |  |  | 12 | 24.5 | 1.00 (Ref) |  |  | 19 | 39.6 | 1.00 (Ref) |  |
| > 50 | 56 | 21.7 |  | 28 | 19.2 | 0.98 (0.43, 2.24) | 0.96 |  | 18 | 13.6 | 0.74 (0.30, 1.82) | 0.51 |  | 3 | 13.3 | 0.39 (0.15, 1.02) | 0.055 |  | 9 | 18.4 | 1.28 (0.39, 4.26) | 0.69 |  | 7 | 14.6 | 0.45 (0.13, 1.56) | 0.21 |

* Sixty-two cases were excluded from analyses because of their missing data for HER2 status or cytokeratin 5/6 and epidermal growth factor receptor status. † Point estimates and 95% confidence intervals were from multivariable models, adjusting for the same series of covariates (except where noticed): age at diagnosis, BMI, age at menarche, age at first pregnancy, number of children, mean breastfeeding duration per child, age at menopause, family history of breast cancer in first degree female relative, occupation, education level, and location of facility. Estimates of numbers of children, cumulative and mean breastfeeding duration, and combined age at first pregnancy and number of children were computed among parous women. ‡ Results were from the trend analysis using the categorical risk factor as a trend. ^a^ Women who reported never pregnant, never gave birth, and had no child were grouped as "Nulliparous" in modeling analyses. ᵇ Multivariable modeling analysis without adjusting for mean breastfeeding duration per child. ^c^ Multivariable modeling analysis without adjusting for age at menopause. ^d^ Analyses restricted to postmenopausal women. AKU, Aga Khan University; BMI, body mass index; CI, confidence interval; HER2, human epidermal growth factor receptor-2; OR, odds ratio; Q, quartile;
