# Supplementary material for: Breast cancer risk factors in relation to molecular subtypes in breast cancer patients from Kenya
Source: Breast Cancer Res. 2021 Jun 26;23:68. doi: 10.1186/s13058-021-01446-3 (PMC8235821; doi:10.1186/s13058-021-01446-3)
Supplement: Supplementary file 7 — Supplementary Table 7.. Associations of key risk factors and tumor subtypes without applying tumor grade to tumor subtype classification [file 13058_2021_1446_MOESM7_ESM.docx]

**Supplementary Table 7. Associations between breast cancer risk factors and tumor molecular subtypes* in Kenyan breast cancer patients (N=821**)**

|  | **Tumor subtypes*** | | | | | | | | | | | | | | | | |
| --- | --- | --- | --- | --- | --- | --- | --- | --- | --- | --- | --- | --- | --- | --- | --- | --- | --- |
|  | **Luminal A n=320** | |  | **Luminal B n=260** | | **Luminal B vs.  Luminal A** | |  | **HER2-enriched  n=88** | | **HER2-enriched vs.  Luminal A** | |  | **Triple Negative  n=153** | | **Triple Negative vs.  Luminal A** | |
|  | **N** | **%** |  | **N** | **%** | **OR (95% CI)†** | ***P†*** |  | **N** | **%** | **OR (95% CI)†** | ***P†*** |  | **N** | **%** | **OR (95% CI)†** | ***P†*** |
| **Age at diagnosis/year** |  |  |  |  |  |  |  |  |  |  |  |  |  |  |  |  |  |
| < 50 | 163 | 51.4 |  | 159 | 61.4 | 1.00 (Ref) |  |  | 44 | 50.0 | 1.00 (Ref) |  |  | 77 | 50.3 | 1.00 (Ref) |  |
| ≥ 50 | 154 | 48.6 |  | 100 | 38.6 | 1.44 (0.60, 3.45) | 0.41 |  | 44 | 50.0 | 2.30 (0.72, 7.33) | 0.16 |  | 76 | 49.7 | 0.65 (0.25, 1.71) | 0.39 |
| **BMI/ kg/m^2^** |  |  |  |  |  |  |  |  |  |  |  |  |  |  |  |  |  |
| Normal (<25.0) | 65 | 26.0 |  | 66 | 32.5 | 1.00 (Ref) |  |  | 29 | 41.4 | 1.00 (Ref) |  |  | 43 | 32.6 | 1.00 (Ref) |  |
| Overweight (25.0 - 29.9) | 100 | 40.0 |  | 83 | 40.9 | 0.91 (0.55, 1.52) | 0.73 |  | 25 | 35.7 | 0.51 (0.26, 1.01) | 0.055 |  | 49 | 37.1 | 0.83 (0.46, 1.52) | 0.55 |
| Obese (≥30.0) | 85 | 34.0 |  | 54 | 26.6 | 0.68 (0.39, 1.20) | 0.18 |  | 16 | 22.9 | **0.35 (0.16, 0.77)** | **0.0096** |  | 40 | 30.3 | 0.86 (0.45, 1.65) | 0.66 |
| Trend‡ |  |  |  |  |  | 0.83 (0.63, 1.10) | 0.19 |  |  |  | **0.58 (0.39, 0.87)** | **0.0082** |  |  |  | 0.93 (0.67, 1.29) | 0.68 |
| **Premenopausal: BMI^a^** |  |  |  |  |  |  |  |  |  |  |  |  |  |  |  |  |  |
| Normal (<25.0) | 40 | 31.5 |  | 47 | 35.9 | 1.00 (Ref) |  |  | 16 | 45.7 | 1.00 (Ref) |  |  | 23 | 38.3 | 1.00 (Ref) |  |
| Overweight (25.0 - 29.9) | 51 | 40.2 |  | 51 | 38.9 | 0.85 (0.45, 1.60) | 0.60 |  | 11 | 31.4 | 0.49 (0.18, 1.29) | 0.15 |  | 16 | 26.7 | 0.46 (0.19, 1.10) | 0.082 |
| Obese (≥30.0) | 36 | 28.4 |  | 33 | 25.2 | 0.74 (0.36, 1.51) | 0.40 |  | 8 | 22.9 | 0.43 (0.14, 1.32) | 0.14 |  | 21 | 35.0 | 0.85 (0.35, 2.03) | 0.71 |
| **Postmenopausal: BMI^a^** |  |  |  |  |  |  |  |  |  |  |  |  |  |  |  |  |  |
| Normal (<25.0) | 25 | 20.3 |  | 19 | 26.8 | 1.00 (Ref) |  |  | 13 | 37.1 | 1.00 (Ref) |  |  | 20 | 27.8 | 1.00 (Ref) |  |
| Overweight (25.0 - 29.9) | 49 | 69.8 |  | 32 | 45.1 | 0.98 (0.41, 2.35) | 0.97 |  | 14 | 40.0 | 0.42 (0.15, 1.21) | 0.11 |  | 33 | 45.8 | 1.49 (0.61, 3.66) | 0.39 |
| Obese (≥30.0) | 49 | 39.8 |  | 20 | 28.2 | 0.81 (0.31, 2.11) | 0.67 |  | 8 | 22.9 | **0.22 (0.06, 0.72)** | **0.013** |  | 19 | 26.4 | 1.14 (0.43, 3.02) | 0.79 |
| Trend‡ |  |  |  |  |  | 0.89 (0.56, 1.44) | 0.64 |  |  |  | **0.45 (0.24, 0.83)** | **0.011** |  |  |  | 1.04 (0.65, 1.67) | 0.87 |
| **Age at menarche/year** |  |  |  |  |  |  |  |  |  |  |  |  |  |  |  |  |  |
| ≤13 (9-13) | 80 | 26.7 |  | 60 | 23.6 | 1.00 (Ref) |  |  | 23 | 26.4 | 1.00 (Ref) |  |  | 33 | 22.9 | 1.00 (Ref) |  |
| 14 | 73 | 24.3 |  | 64 | 25.2 | 1.47 (0.83, 2.60) | 0.18 |  | 26 | 29.9 | 1.74 (0.77, 3.90) | 0.18 |  | 37 | 25.7 | 1.49 (0.77, 2.89) | 0.24 |
| ≥15 (15-20) | 147 | 49.0 |  | 130 | 51.2 | 1.38 (0.83, 2.28) | 0.21 |  | 38 | 43.7 | 1.33 (0.65, 2.74) | 0.44 |  | 74 | 51.4 | 1.44 (0.80, 2.59) | 0.23 |
| **Age at first pregnancy/year** |  |  |  |  |  |  |  |  |  |  |  |  |  |  |  |  |  |
| <20 | 76 | 24.6 |  | 54 | 20.9 | 1.00 (Ref) |  |  | 31 | 35.6 | 1.00 (Ref) |  |  | 51 | 34.0 | 1.00 (Ref) |  |
| 20-24 | 139 | 45.0 |  | 115 | 44.6 | 1.13 (0.63, 2.03) | 0.68 |  | 30 | 34.5 | 0.55 (0.26, 1.19) | 0.13 |  | 69 | 46.0 | 0.71 (0.38, 1.31) | 0.27 |
| 25-29 | 62 | 20.1 |  | 51 | 19.8 | 1.12 (0.54, 2.33) | 0.76 |  | 16 | 18.4 | 0.74 (0.28, 2.00) | 0.55 |  | 19 | 12.7 | 0.54 (0.23, 1.27) | 0.16 |
| Nulliparous^b^ or ≥30 | 32 | 10.4 |  | 38 | 14.7 | 1.05 (0.39, 2.78) | 0.93 |  | 10 | 11.5 | 1.21 (0.32, 4.61) | 0.78 |  | 11 | 7.3 | 0.68 (0.21, 2.21) | 0.52 |
| **Parity** |  |  |  |  |  |  |  |  |  |  |  |  |  |  |  |  |  |
| Nulliparous^b^ | 305 | 95.3 |  | 241 | 92.7 | 0.67 (0.11, 4.02) | 0.97 |  | 4 | 4.5 | 0.56 (0.07, 4.63) | 0.59 |  | 3 | 2.0 | 0.67 (0.11, 4.02) | 0.66 |
| Parous | 15 | 4.7 |  | 19 | 7.3 | 1.00 (Ref) |  |  | 84 | 95.5 | 1.00 (Ref) |  |  | 150 | 98.0 | 1.00 (Ref) |  |
| **Number of children** |  |  |  |  |  |  |  |  |  |  |  |  |  |  |  |  |  |
| 1 or 2 | 84 | 27.5 |  | 90 | 37.3 | 1.00 (Ref) |  |  | 19 | 22.6 | 1.00 (Ref) |  |  | 31 | 20.7 | 1.00 (Ref) |  |
| 3 or 4 | 122 | 40.0 |  | 92 | 38.2 | **0.52 (0.31, 0.87)** | **0.013** |  | 26 | 31.0 | 0.93 (0.41, 2.15) | 0.87 |  | 59 | 39.3 | 1.29 (0.66, 2.50) | 0.46 |
| ≥ 5 | 99 | 32.5 |  | 59 | 24.5 | **0.37 (0.19, 0.73)** | **0.0040** |  | 39 | 46.4 | 1.68 (0.64, 4.36) | 0.29 |  | 60 | 40.0 | 1.26 (0.57, 2.80) | 0.57 |
| Trend‡ |  |  |  |  |  | **0.60 (0.43, 0.84)** | **0.0025** |  |  |  | 1.33 (0.82, 2.16) | 0.26 |  |  |  | 1.11 (0.75, 1.64) | 0.61 |
| **Cumulative breastfeeding**  **duration/month^c^** |  |  |  |  |  |  |  |  |  |  |  |  |  |  |  |  |  |
| Q1: 1 - <39 | 78 | 26.5 |  | 74 | 31.6 | 1.00 (Ref) |  |  | 15 | 18.3 | 1.00 (Ref) |  |  | 20 | 13.9 | 1.00 (Ref) |  |
| Q2: 39 - <62 | 66 | 22.5 |  | 61 | 26.1 | 1.19 (0.66, 2.16) | 0.57 |  | 22 | 26.8 | **2.72 (1.08, 6.86)** | **0.034** |  | 40 | 27.8 | **3.27 (1.48, 7.23)** | **0.0035** |
| Q3: 62 - <96 | 76 | 25.9 |  | 48 | 20.5 | 0.80 (0.40, 1.61) | 0.54 |  | 14 | 17.1 | 0.90 (0.30, 2.66) | 0.85 |  | 40 | 27.8 | 2.03 (0.85, 4.88) | 0.11 |
| Q4: 96 - 720 | 74 | 25.2 |  | 51 | 21.8 | 1.38 (0.61, 3.13) | 0.44 |  | 31 | 37.8 | 1.49 (0.47, 4.73) | 0.50 |  | 44 | 30.6 | 2.15 (0.80, 5.75) | 0.13 |
| Trend‡ |  |  |  |  |  | 1.03 (0.79, 1.34) | 0.83 |  |  |  | 1.02 (0.71, 1.47) | 0.91 |  |  |  | 1.18 (0.87, 1.59) | 0.29 |
| **Mean breastfeeding duration**  **per child/month** |  |  |  |  |  |  |  |  |  |  |  |  |  |  |  |  |  |
| <12 | 44 | 15.0 |  | 38 | 16.2 | 1.00 (Ref) |  |  | 11 | 13.4 | 1.00 (Ref) |  |  | 23 | 16.0 | 1.00 (Ref) |  |
| 12- 23 | 154 | 52.4 |  | 127 | 54.3 | 0.82 (0.44, 1.53) | 0.52 |  | 49 | 59.8 | 1.42 (0.54, 3.68) | 0.48 |  | 71 | 49.3 | 0.86 (0.42, 1.78) | 0.68 |
| ≥ 24 | 96 | 32.7 |  | 69 | 29.5 | 0.72 (0.36, 1.42) | 0.34 |  | 22 | 26.8 | 1.31 (0.46, 3.75) | 0.61 |  | 50 | 34.7 | 1.19 (0.55, 2.58) | 0.66 |
| Trend‡ |  |  |  |  |  | 0.85 (0.61, 1.19) | 0.34 |  |  |  | 1.09 (0.67, 1.75) | 0.73 |  |  |  | 1.15 (0.79, 1.68) | 0.47 |
| **Age at first pregnancy &**  **Number of children** |  |  |  |  |  |  |  |  |  |  |  |  |  |  |  |  |  |
| Age 25+ yr, 1-3 births | 64 | 21.8 |  | 54 | 22.6 | 1.00 (Ref) |  |  | 15 | 17.9 | 1.00 (Ref) |  |  | 20 | 13.6 | 1.00 (Ref) |  |
| Age <25 yr, 1-3 births | 86 | 29.3 |  | 87 | 36.4 | 1.16 (0.65, 2.09) | 0.61 |  | 19 | 22.6 | 0.92 (0.35, 2.37) | 0.86 |  | 47 | 32.0 | 1.55 (0.73, 3.27) | 0.25 |
| Age 25+ yr, 4+ births | 16 | 5.4 |  | 17 | 7.1 | 0.92 (0.34, 2.46) | 0.87 |  | 8 | 9.5 | 2.30 (0.65, 8.14) | 0.20 |  | 7 | 4.8 | 1.23 (0.33, 4.61) | 0.76 |
| Age <25 yr, 4+ births | 128 | 43.5 |  | 81 | 33.9 | 0.54 (0.28, 1.03) | 0.061 |  | 42 | 50.0 | 1.35 (0.53, 3.44) | 0.53 |  | 73 | 49.7 | 1.43 (0.65, 3.14) | 0.37 |
| Trend‡ |  |  |  |  |  | **0.78 (0.64, 0.96)** | **0.020** |  |  |  | 1.15 (0.86, 1.55) | 0.34 |  |  |  | 1.07 (0.84, 1.36) | 0.57 |
| **Number of children & Cumulative breastfeeding duration** |  |  |  |  |  |  |  |  |  |  |  |  |  |  |  |  |  |
| Nulliparous or ≤3 children &  <62 months | 132 | 42.7 |  | 131 | 51.8 | 1.00 (Ref) |  |  | 31 | 36.0 | 1.00 (Ref) |  |  | 51 | 34.7 | 1.00 (Ref) |  |
| ≤3 children & ≥62 months | 31 | 10.0 |  | 24 | 9.5 | 0.68 (0.34, 1.37) | 0.29 |  | 6 | 7.0 | 0.45 (0.12, 1.69) | 0.24 |  | 17 | 11.6 | 1.29 (0.58, 2.87) | 0.53 |
| ≥4 children & <62 months | 27 | 8.7 |  | 23 | 9.1 | 0.64 (0.27, 1.50) | 0.30 |  | 10 | 11.6 | 2.03 (0.70, 5.85) | 0.19 |  | 12 | 8.2 | 1.14 (0.44, 2.95) | 0.78 |
| ≥4 children & ≥62 months | 119 | 38.5 |  | 75 | 29.6 | **0.49 (0.28, 0.85)** | **0.011** |  | 39 | 45.3 | 1.28 (0.59, 2.79) | 0.53 |  | 67 | 45.6 | 0.98 (0.52, 1.85) | 0.96 |
| Trend‡ |  |  |  |  |  | **0.79 (0.66, 0.94)** | **0.0096** |  |  |  | 1.11 (0.86, 1.44) | 0.43 |  |  |  | 0.99 (0.80, 1.22) | 0.93 |
| **Menopausal status^a^** |  |  |  |  |  |  |  |  |  |  |  |  |  |  |  |  |  |
| Premenopausal | 163 | 50.9 |  | 155 | 59.9 | 1.00 (Ref) |  |  | 44 | 50.0 | 1.00 (Ref) |  |  | 68 | 44.7 | 1.00 (Ref) |  |
| Postmenopausal | 157 | 49.1 |  | 104 | 40.2 | 0.53 (0.25, 1.16) | 0.11 |  | 44 | 50.0 | 0.64 (0.21, 1.92) | 0.42 |  | 84 | 55.3 | 1.68 (0.74, 3.85) | 0.22 |
| **Age at menopause/year** |  |  |  |  |  |  |  |  |  |  |  |  |  |  |  |  |  |
| Premenopausal | 163 | 50.9 |  | 155 | 59.6 | *N/A* | |  | 44 | 50.0 | *N/A* | |  | 68 | 44.4 | *N/A* | |
| < 50 years | 61 | 21.1 |  | 55 | 22.3 | 1.00 (Ref) |  |  | 28 | 33.7 | 1.00 (Ref) |  |  | 44 | 32.1 | 1.00 (Ref) |  |
| > 50 years | 65 | 22.5 |  | 37 | 15.0 | 0.62 (0.31, 1.25) | 0.18 |  | 11 | 13.3 | **0.34 (0.13, 0.88)** | **0.026** |  | 25 | 18.3 | 0.59 (0.28, 1.23) | 0.16 |

* Tumor subtypes were determined without using tumor grade in the absence of ki67. ** Seventeen cases were excluded from analyses because of their missing data for HER2 status. † Point estimates and 95% confidence intervals were from multivariable models, adjusting for the same series of covariates (except where noticed): age at diagnosis, BMI, age at menarche, age at first pregnancy, number of children, mean breastfeeding duration per child, age at menopause, family history of breast cancer in first degree female relative, occupation, education level, and location of facility. Estimates of numbers of children, cumulative and mean breastfeeding duration, and combined age at first pregnancy and number of children were computed among parous women. ‡ Results were from the trend analysis using the categorical risk factor as a trend. ᵃ Multivariable modeling analysis without adjusting for age at menopause. ᵇ Women who reported never pregnant, never gave birth, and had no child were grouped as "Nulliparous" in modeling analyses. ^c^ Multivariable modeling analysis without adjusting for mean breastfeeding duration per child. BMI, body mass index; CI, confidence interval; HER2, human epidermal growth factor receptor-2; OR, odds ratio; Q, quartile.
