# Supplementary material for: Breast cancer risk factors in relation to molecular subtypes in breast cancer patients from Kenya
Source: Breast Cancer Res. 2021 Jun 26;23:68. doi: 10.1186/s13058-021-01446-3 (PMC8235821; doi:10.1186/s13058-021-01446-3)
Supplement: Supplementary file 9 — Supplementary Table 9.. Associations between age at first pregnancy and ER status .in parous women [file 13058_2021_1446_MOESM9_ESM.docx]

| **Supplementary Table 9. Sensitivity analyses: Associations between age at first pregnancy and receptor status among parous women** | | | | | | |
| --- | --- | --- | --- | --- | --- | --- |
|  | **ER⁺ N=531** | | **ER⁻ N=246** | | **ER⁻ vs. ER+** | |
|  | **N** | **%** | **N** | **%** | **OR (95% CI)†** | ***P†*** |
| **Age at first pregnancy/year** |  |  |  |  |  |  |
| <20 | 129 | 24.3 | 86 | 35.0 | 1.00 (Ref) |  |
| 20-24 | 251 | 47.3 | 108 | 43.9 | 0.68 (0.42, 1.10) | 0.12 |
| ≥25 | 151 | 28.4 | 52 | 21.1 | 0.64 (0.35, 1.19) | 0.16 |
| Trend‡ |  |  |  |  | 0.80 (0.59, 1.09) | 0.16 |

† Point estimates and 95% confidence intervals were from multivariable models, adjusting for the same series of covariates: age at diagnosis, BMI, age at menarche, age at first pregnancy, number of children, mean breastfeeding duration per child, age at menopause, family history of breast cancer in first degree female relative, occupation, education level, and location of facility. ‡ Results were from the trend analysis using the categorized age at first pregnancy as a trend. CI, confidence interval; ER, estrogen receptor; OR, odds ratio.
