# Supplementary material for: Breast cancer risk factors in relation to molecular subtypes in breast cancer patients from Kenya
Source: Breast Cancer Res. 2021 Jun 26;23:68. doi: 10.1186/s13058-021-01446-3 (PMC8235821; doi:10.1186/s13058-021-01446-3)
Supplement: Supplementary file 10 — Supplementary Figure 1.. Associations between BMI and HER2 status stratified by hospital groups [file 13058_2021_1446_MOESM10_ESM.docx]

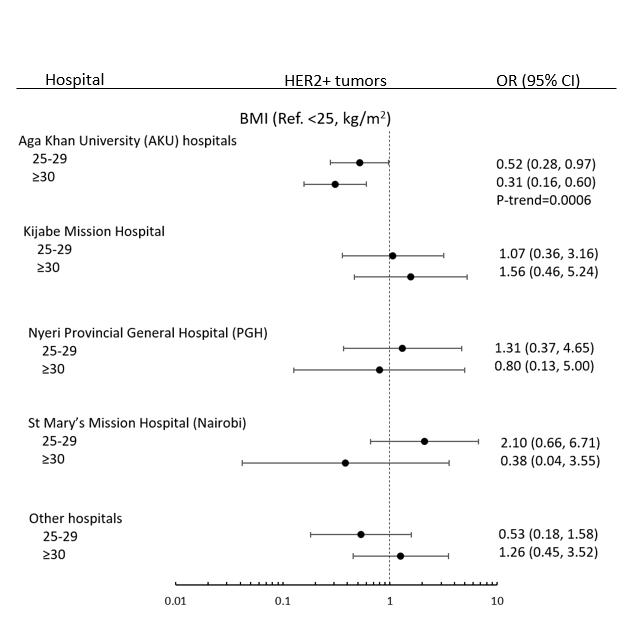


**Supplementary Figure 1. Associations between BMI and HER2 status stratified by hospital groups.** Odds ratios (OR) and 95% confidence interval (CI) were calculated from multivariable logistic regression models after adjusting for categorized age at diagnosis.
